# Two-dimensional infrared spectroscopy as a tool to reveal the vibrational and molecular structure of [FeFe] hydrogenases†

**DOI:** 10.1039/d5sc01811k

**Published:** 2025-05-07

**Authors:** Cornelius C. M. Bernitzky, Yvonne Rippers, Denise Poire, Mathesh Vaithiyanathan, Solomon L. D. Wrathall, Barbara Procacci, Igor V. Sazanovich, Gregory M. Greetham, Patricia Rodríguez-Macía, Neil T. Hunt, James A. Birrell, Marius Horch

**Affiliations:** a Freie Universität Berlin, Department of Physics, Ultrafast Dynamics in Catalysis Arnimallee 14 14195 Berlin Germany marius.horch@fu-berlin.de; b Technische Universität Berlin, Department of Chemistry, Modelling of Biomolecular Systems Straße des 17. Juni 135 10623 Berlin Germany; c Department of Chemistry and York Biomedical Research Institute, University of York York YO10 5DD UK; d STFC Central Laser Facility, Research Complex at Harwell, Rutherford Appleton Laboratory Harwell Campus Didcot OX11 0QX UK; e School of Chemistry and Leicester Institute for Structural and Chemical Biology, University of Leicester, University Road Leicester LE1 7RH UK; f School of Life Sciences, University of Essex Wivenhoe Park Colchester CO4 3SQ UK

## Abstract

[FeFe] hydrogenases are Nature's most efficient catalysts for the cleavage and evolution of molecular hydrogen. Despite decades of research, key aspects of the catalytic cycle and the underlying geometrical and electronic properties of the active-site cofactor, called the H-cluster, are not fully understood. Spectroscopic techniques have played a central role in establishing the current state of knowledge on [FeFe] hydrogenases, and further advances in the field depend critically on novel techniques that yield so-far inaccessible insights into structural and mechanistic aspects. Infrared (IR) absorption spectroscopy represents a well-established and versatile technique that can identify and characterize all active and inactive states of the H-cluster by means of structurally sensitive and spectrally isolated CO and CN stretching vibrations. However, the amount of information that can be extracted from these linear experiments is inherently limited. Here we introduce experimental and computational two-dimensional (2D-)IR spectroscopy for the characterization of [FeFe] hydrogenases. Utilizing the H_inact_ state of the H-cluster as a model system, we demonstrate that this nonlinear technique yields direct information about the nature and interactions of the CO and CN stretching vibrations. These insights allow, for the first time, to quantitatively describe the character of these widely used reporter vibrations, their spatial localization, and the way they change upon structural variation of the H-cluster. The strength of this approach is demonstrated by correctly identifying the proposed structure of the H_inact_ state, in solution and at ambient temperature. In conclusion, the introduced combination of experimental and computational 2D-IR spectroscopy represents a powerful approach for studying [FeFe] hydrogenases and other complex organometallic targets.

## Abstract 2

In this proof-of-concept study, we demonstrate how experimental and computational 2D-IR spectroscopy can provide insights into the vibrational and molecular structure of [FeFe] hydrogenases.

## Graphical abstract

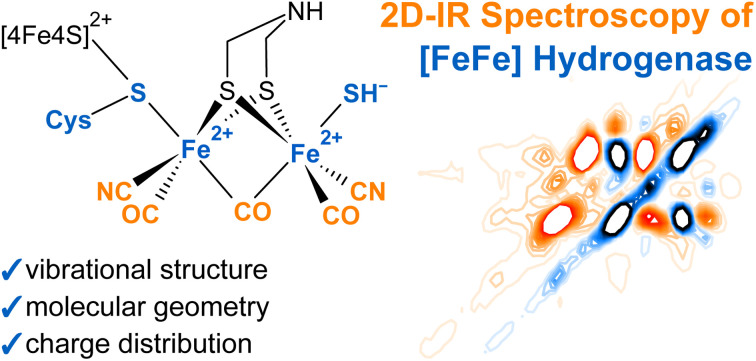

## Introduction

Hydrogenases are complex metalloenzymes that catalyse the reversible cleavage of molecular hydrogen (H_2_) into protons and electrons. Thus, they are valuable model systems for sustainable energy conversion approaches utilizing H_2_ as a clean fuel.^1^ In this respect, the bioinspired design of synthetic catalysts for H_2_ activation and evolution represents a particularly promising strategy given that a detailed understanding of the structural, dynamic, and mechanistic features of the biological blueprints is available. Thus, physical techniques that provide insights into these aspects have played a key role in molecular hydrogenase research since the very beginning.^2^

**Fig. 1:**
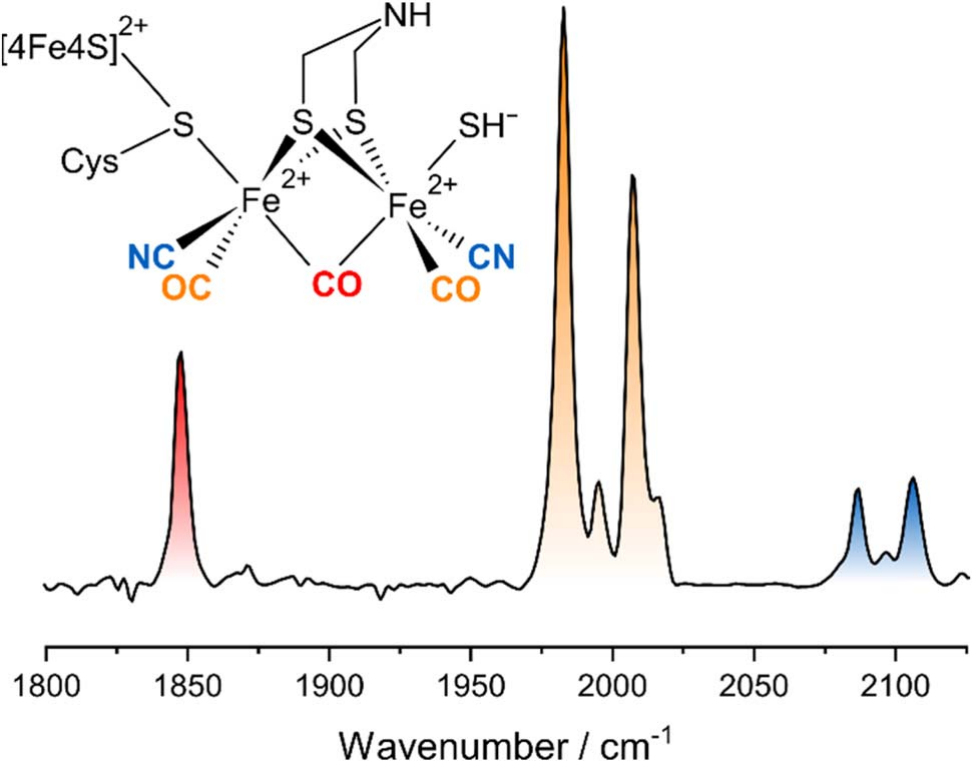
Schematic representation and linear IR absorption spectrum of the H-cluster in the H_inact_ state. The IR spectrum was recorded from *Dd*HydAB.

Hydrogenases can be classified according to the metal content of their catalytic sites.^2^ Besides [Fe] hydrogenases, which are hydride-transferring enzymes featuring a monometallic active site, two bimetallic classes can be distinguished, both of which catalyse the interconversion of H_2_ with protons and electrons. [NiFe] hydrogenases harbour a heterobimetallic active site containing Ni and Fe that is linked to the protein *via* a chelating scaffold of four cysteines, thereby facilitating the formation of entatic states.^3,4^ By contrast, [FeFe] hydrogenases utilize a homobimetallic active site containing two Fe ions in formal oxidation states of +1 or +2 (Fig. 1).^5^ This [2Fe] centre contains a unique 2-azapropane-1,3-dithiolate (ADT) ligand, which bridges the two iron ions and can be protonated at the central bridgehead atom. The unique metal centre is covalently linked to the protein *via* a cysteine ligand that coordinates both the [2Fe] unit and a cubane [4Fe–4S] cluster (Fig. 1). The two iron ions of the [2Fe] centre are generally called Fe_d_ (distal) and Fe_p_ (proximal), based on their distance to the [4Fe–4S] cluster. The entirety of the [2Fe] centre (called [2Fe]_H_) and the [4Fe–4S] cluster (called [4Fe–4S]_H_) is generally denoted as the ‘H-cluster’. All classes of hydrogenases also feature biologically uncommon diatomic ligands bound to Fe, *i.e.* carbon monoxide (CO) and cyanide (CN^−^).^6–12^ One CO and two CN^−^ ligands coordinate the single Fe ion of [NiFe] hydrogenase,^6–8,10–12^ while a total of three CO and two CN^−^ are distributed among the two Fe sites of [2Fe]_H_ in [FeFe] hydrogenases.^9,10^ Specifically, Fe_d_ and Fe_p_ carry one terminal CO and one terminal CN^−^ ligand each, while the third CO ligand bridges the two Fe ions (Fig. 1). This architecture leaves a vacant coordination site on Fe_d_, which can be occupied by substrate-derived ligands or inhibitors. For instance, a mono-sulfur ligand, assigned as HS^−^, can be found in an oxygen-stable inhibited state called H_inact_ (Fig. 1), which features a di-ferrous [2Fe] centre (Fe^+2^Fe^+2^) and an oxidized [4Fe–4S]^2+^ cluster.^13^

Besides tuning the functional properties of the catalytic sites of hydrogenases, diatomic CO and CN^−^ ligands can also be utilized as highly localized reporter groups for infrared (IR) spectroscopy.^6–12,14–16^ Specifically, these IR chromophores give rise to well-defined, spectrally isolated, and structurally sensitive *ν*(CO) and *ν*(CN) stretching vibrations with large transition dipole moments (Fig. 1). As a consequence, IR spectroscopy has been used to study the structure and mechanism of hydrogenases since the discovery of active-site CO and CN^−^ ligands by this very technique.^6–12,14–16^ While IR spectroscopy is a particularly flexible and biocompatible technique, the amount of structural and dynamical information that can be extracted from linear IR absorption experiments is inherently limited. To overcome this limitation, we have recently introduced third-order nonlinear IR techniques to hydrogenase research.^4,17–20^ In simple terms, these nonlinear techniques differ from a linear absorption experiment in the type and number of light–matter interactions.^21^ A conventional IR absorption experiment involves a single incoming interaction of the sample with the continuous IR beam from an ordinary probe light source. The amount of absorbed probe light scales linearly with the incident intensity, and the result is an absorbance spectrum. By contrast, nonlinear IR techniques involve multiple interactions of the sample with ultrashort IR laser pulses that lead to a sequence of vibrational transitions. The molecular response does not scale linearly with the total incident intensity, and the resultant spectrum depends on the pulse sequence. With one pump and one probe pulse, an IR_pump_–IR_probe_ spectrum is obtained that can be understood as the change in probe light absorbance due to the previous interaction of the sample with the pump. Adding a second, variably delayed pump pulse represents one way to record a two-dimensional (2D-)IR spectrum, which resolves the IR_pump_–IR_probe_ spectrum in terms of the pump frequency. More details about the 2D-IR technique and the technical implementation used here can be found in the literature.^21–25^

Utilizing an O_2_-tolerant [NiFe] hydrogenase as a simple and well-explored model system, we have demonstrated how ultrafast 2D-IR spectroscopies can be used to gain detailed insights into, *e.g.*, bond properties, intramolecular interactions, structural dynamics, and ultrafast energy transfer.^4^ This proof-of-concept study was later extended to computational spectroscopy^18^ as well as further experimental work on other [NiFe] hydrogenases^17,20^ and a subsite analogue of the active-site Fe(CO)(CN^−^)_2_ moiety of these enzymes.^19^

Here, we apply experimental and computational 2D-IR spectroscopy to the more complex [FeFe] hydrogenases, thereby expanding our work beyond [NiFe] hydrogenases^4,17–20^ and previous 2D-IR studies on synthetic model complexes mimicking the [2Fe]_H_ moiety.^26,27^ Utilizing the H_inact_ state of the [FeFe] hydrogenase from *Desulfovibrio desulfuricans* (*Dd*HydAB) as an oxygen-stable model species that can be enriched to high purity,^13,28^ we will demonstrate that these techniques provide detailed insights into the Fe_2_(CO)_3_(CN^−^)_2_ vibrational manifold as well as the vibrational and molecular structure of the [2Fe] active-site moiety of these enzymes. *Inter alia*, this information allows understanding of how the localization and coupling of *ν*(CO) and *ν*(CN) stretching vibrations is linked to the symmetry of the active site. Besides immediate insights into molecular structure and charge distribution, this information also provides the opportunity to utilize all information accessible from vibrational calculations to validate structural models of catalytic intermediates.

## Experimental and computational procedures

### Ultrafast 2D-IR spectroscopy

The H_inact_ state of *Dd*HydAB (*ca.* 2 mM) was prepared as described previously.^13^ All spectra were recorded in transmission mode using a gas-tight and temperature-controlled (*T* = 283 K) small-volume sandwich cell (optical path length = 50 μm, *V* ≈ 8 μL) equipped with CaF_2_ windows. All data were acquired in (pseudo) pump–probe geometry utilizing mid-IR pulses (centre frequency = 1950 cm^−1^; bandwidth > 300 cm^−1^; pulse duration = 50 fs; repetition rate = 10 kHz; pump energy: 1 μJ; probe energy: 100 nJ) from the ULTRA laser-system as described previously.^22–24,29^

2D-IR spectra (accumulation time = 300 s) were obtained with parallel and perpendicular pump–probe polarization at a waiting time of *T*_w_ = 250 fs. The 2D-IR data were obtained in a time-domain fashion by scanning the coherence time *τ* between two pulse-shaper generated collinear pump pulses from 0 to 6 ps (step size 30 fs) prior to overlap with the probe pulse and self-heterodyned detection of the collinearly emitted signal.^22–24^ The pump frequency axis was obtained by Fourier transformation of the time-domain signal with respect to *τ* (spectral resolution ≈ 2.8 cm^−1^), while the probe frequency axis was obtained by signal dispersion in two spectrographs and detection *via* two liquid–nitrogen cooled 128-element MCT detectors (spectral resolution < 2.5 cm^−1^). Four-frame phase cycling was employed in 2D-IR data acquisition to limit contributions from pump light scattered on the detector.^23^

### Quantum chemical calculations

Quantum chemical calculations were performed in Gaussian16 (revision A.13) on the density functional level of theory.^30^ The BP86 functional was employed in all cases,^31^ using the triple-zeta valence basis set including polarization functions, def2-tzvp,^32^ for the Fe ions and the 6-31g(d) split-valence double-zeta basis set for all other atoms.^33^ Further computational choices were made with the high-accuracy demands of anharmonic frequency analyses in mind (*vide infra*).^18^ A ‘superfine’ pruned integration grid was used for computing two-electron integrals and their derivatives throughout all calculations (175/250 radial shells for first-row/higher-row atoms and 974 points per shell in both cases). The convergence criteria for the self-consistent-field procedure (root-mean-square and maximum change of the density matrix) were set to 10^−9^ and 10^−7^, respectively. According to the Gaussian16 documentation, this corresponds to an energy change of 10^−18^ Ha.

Starting geometries for all computational models were constructed from the crystal structure of the H_inact_ state of *Dd*HydAB (PDB entry 6SG2).^13^ Unless indicated otherwise, all models included the two Fe ions of the [2Fe] center, the bridging ADT ligand, and the diatomic CO/CN^−^ ligands. Hydrogen atoms were added appropriately, and the apical ligands of the two Fe sites were varied systematically, as described in the Results and discussion section. For models containing neutral cysteine (Cys–SH) as an apical ligand on the proximal iron, the proton was modelled as a Triton (^3^H^+^) to avoid the possible formation of normal modes with S–H stretching coordinates of both the cysteine and the SH^−^ ligand at the distal iron (*e.g.* symmetric and antisymmetric combinations). The same strategy was also explored for models with apical SH^−^ and H_2_S ligands at the proximal iron. For the former, the spectroscopic properties of both isotopologues were practically indistinguishable, while for the latter, an artificial mixing of some normal modes was observed. As a consequence, both SH^−^ and H_2_S ligands were modelled with ordinary protons (^1^H^+^). A closed-shell di-ferrous (Fe^+2^Fe^+2^) singlet ground state was assumed for all models (*S* = 0, *M* = 1). Geometries were fully optimized using an analytic Hessian matrix in the final optimization steps and ‘very tight’ convergence criteria (maximum force = 2.0 × 10^−6^, root-mean-square force = 1.0 × 10^−6^, maximum displacement = 6.0 × 10^−6^, and root-mean-square displacement = 4.0 × 10^−6^; all in atomic units).

Harmonic vibrational frequencies were calculated by diagonalization of the mass-weighted Hessian matrix. Anharmonic transition energies of fundamental, overtone, and combination transitions were calculated using an automated implementation of the generalized second-order vibrational perturbation theory (GVPT2) approach.^34,35^ Cubic and semi-diagonal quartic force constants for these analyses were obtained by numerical derivation of analytic Hessian matrices with respect to the normal coordinates. Individual low-frequency modes that were found to be problematic in the treatment of resonances were ‘inactivated’, *i.e.* they were not included in the final GVPT2 analysis, but associated cubic and quartic force constants were retained.^35^

Harmonic and anharmonic fundamental frequencies were directly extracted from the Gaussian output. Frequencies of sequence transitions between excited vibrational levels were calculated from anharmonic fundamental, overtone, and combination transition frequencies as described previously.^18^ The potential energy distribution of the individual normal modes was evaluated using a home-made program.^36^

## Results and discussion

**Fig. 2:**
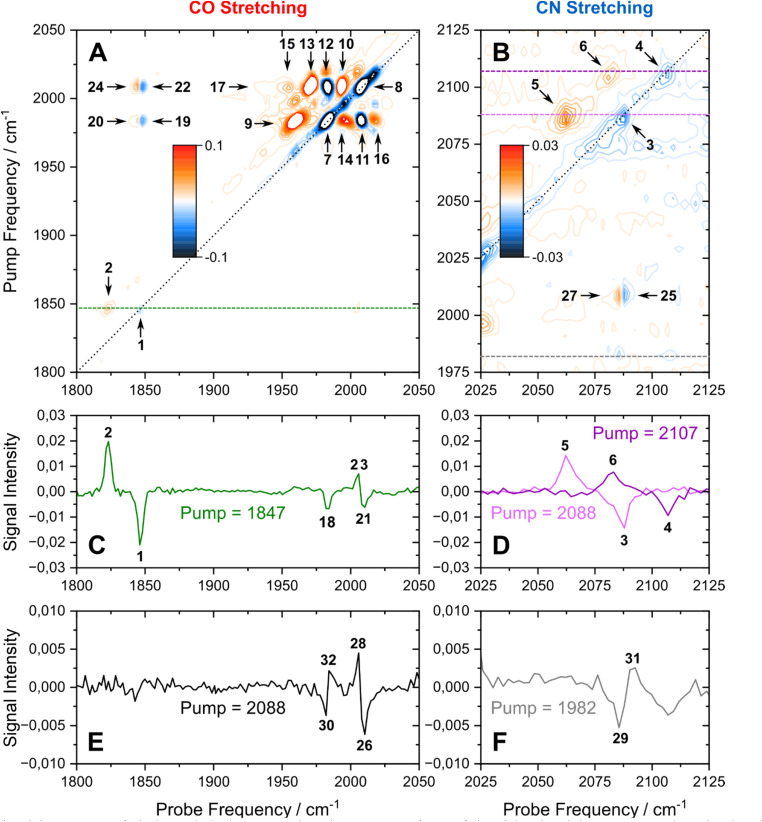
(A and B) 2D-IR spectrum of *Dd*HydAB enriched in the H_inact_ state, obtained at a waiting time of *T*_w_ = 250 fs. (C to F) Slices through the 2D-IR spectrum taken at the indicated pump frequencies. All data were recorded with perpendicular polarization of pump and probe pulses. Signals are labelled according to the main text. Also see Fig. S1–S5.†

**Fig. 3:**
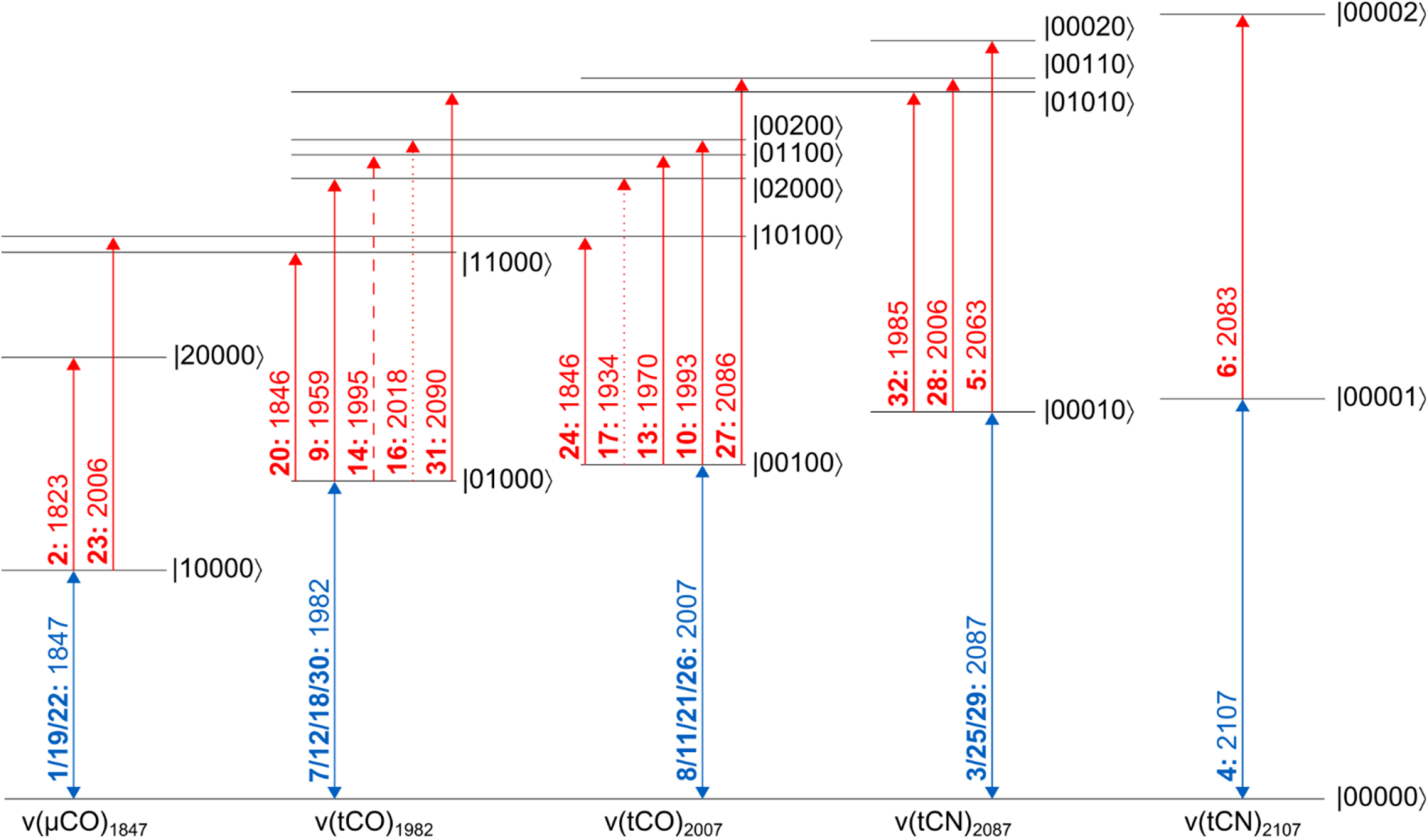
Energy level scheme illustrating the vibrational transitions probed in this study. All energy eigenstates are labelled as |abcde〉, where each entry refers to the number of vibrational quanta in each one of the five vibrational modes (listed by increasing frequency). Dipole-forbidden transitions are shown as dotted lines. A weakly resolved transition is depicted as a dashed line. Signals are labelled as bold numbers according to the main text. All transitions energies, given in units of cm^−1^, correspond to the probe frequencies of the indicated signals.

The linear IR absorption spectrum of [FeFe] hydrogenases exhibits a set of five signals in the spectral range between *ca.* 1800 and 2100 cm^−1^ for each redox-structural state of the H-cluster. While the H_inact_ state can be prepared to hight purity, some minor contributions from other states can be observed in both linear and nonlinear IR spectra. These will not be discussed in the following. For the H_inact_ state of *Dd*HydAB, a pattern of five signals at ground-state absorption (**0-1**) frequencies of *ca.* 1847, 1983, 2007, 2086, and 2106 cm^−1^ (Fig. 1) can be detected, as reported previously.^13^ In line with studies on other H-cluster intermediates, the medium-sized signal at 1847 cm^−1^ (red) can be approximated as a stretching vibration of the bridging CO ligand, *ν*(μCO), while the two dominant signals at 1983 and 2007 cm^−1^ (orange) predominantly reflect stretching vibrations of the two terminal CO ligands, *ν*(tCO). Finally, the high-frequency doublet at 2086 and 2106 cm^−1^ (blue) corresponds to the stretching vibrations of the two cyanide ligands, *ν*(tCN). These three sets of ligand stretching vibrations are frequently used as structural markers or for identifying redox-structural states of the H-cluster. However, the exact nature of these reporter vibrations, the interactions between them, and the encoded structural information cannot be easily deduced from the linear IR absorption spectrum. Thus, we turn to the 2D-IR spectrum of the H_inact_ state of *Dd*HydAB, recorded at a waiting time of *T*_W_ = 250 fs (Fig. 2), to gain insights into these aspects. For the sake of simplicity and consistency with earlier studies, signals in this spectrum will be generally explained by using pump–probe terminology. All vibrational transitions and the corresponding energies are numbered and illustrated in Fig. 3. All discussed peak positions were obtained by fitting Gaussian lineshape functions to pump slices through the 2D-IR spectrum (Fig. S1–S5†).

Signals in a 2D-IR spectrum can be roughly grouped into two categories. On the one hand, signals on or close to the diagonal of the two-dimensional spectrum usually reflect transitions that can be associated with a single vibrational mode (diagonal peaks). On the other hand, signals in the off-diagonal region reflect interactions between different vibrational degrees of freedom (cross peaks). We start our analysis of the H_inact_ state of *Dd*HydAB by inspecting the diagonal peaks.

According to the IR absorption spectrum, we expect diagonal signals in three well-separated frequency regions that can be assigned to one *ν*(μCO) as well as two *ν*(tCO) and two *ν*(tCN) vibrations. In the low-frequency part of the 2D-IR spectrum, a negative signal **1** can be observed at pump/probe frequency of 1847 cm^−1^ (Fig. 2A and C). This on-diagonal signal, coinciding with the **0-1** transition frequency of the *ν*(μCO) mode, corresponds to a bleaching of the vibrational ground state and stimulated emission from the first excited vibrational level. Pumping at 1847 cm^−1^ also yields a positive signal **2** at a slightly lower probe frequency of 1823 cm^−1^ (Fig. 2A and C), which can be assigned to excited-state absorption from the first to the second vibrational level of this mode (**1-2**). The difference between **0-1** and **1-2** transition frequencies corresponds to the intramode anharmonicity, which reflects the deviation of the vibrational potential from the idealized parabolic shape expected for a harmonic oscillator. The obtained value of 24 cm^−1^ agrees well with values reported for the *ν*(CO) mode of [NiFe] hydrogenases and other mono-carbonyl compounds.^4,17,19,20,37^ This observation demonstrates that the *ν*(μCO)_1847_ mode of the H_inact_ state can be interpreted as a bond-localized vibration of the bridging μCO ligand of the H-cluster. This finding agrees with the large frequency separation of this mode from any of the other *ν*(CX) vibrational modes.

The high frequency part of the 2D-IR spectrum exhibits two on-diagonal signals, **3** and **4**, at pump/probe frequencies of 2088/2087 and 2107 cm^−1^, respectively (Fig. 2B and D). These signals can be assigned to ground-state bleaching and stimulated emission (**0-1**) of the two *ν*(tCN) modes. Corresponding positive signals **5** and **6**, reflecting excited-state absorption (**1-2**), are observed at 2088/2063 and 2107/2083 cm^−1^, respectively (Fig. 2B and D). Together, these values yield intramode anharmonicities of 24 cm^−1^ for both *ν*(tCN) modes. Again, these values are consistent with largely bond-localized vibrations, *i.e.* the two *ν*(tCN) modes are not well described as symmetric and antisymmetric combinations, in contrast to the situation in [NiFe] hydrogenases.^12,18^ This interpretation is supported by the absence of detectable cross peaks connecting the two on-diagonal signals (no bleaches at pump/probe frequencies of 2088/2107 and 2107/2087 cm^−1^; Fig. 2B and D). Interestingly, bond-localized *ν*(tCN) vibrations were previously proposed for other redox-structural states, based on linear IR absorption studies on amino-acid exchange variants of two different [FeFe] hydrogenases.^38^ Similar conclusions were drawn based on 2D-IR studies on an [FeFe] model complex.^27^ Our findings provide the first direct proof for this hypothesis, and the introduced analysis yields immediate access to this type of information without requiring a manipulation of the native protein structure of the enzyme.

After inspecting weaker signals at lower and higher *ν*(CX) spectral regions, we next focus on the most pronounced signals in the central part of the 2D-IR spectrum. We observe two negative signals **7** and **8** on the diagonal at pump/probe frequencies of 1982 and 2007 cm^−1^ (Fig. 2A). These can be assigned to ground-state bleaching and stimulated emission (**0-1**) of the two *ν*(tCO) modes. Moreover, and in contrast to observations for *ν*(μCO) and *ν*(tCN) modes, a complex pattern of additional signals is observed in the spectral region typical for the *ν*(tCO) modes. Specifically, we detect multiple signals at pump frequencies that correspond to the fundamental **0-1** transition of these modes (1982 and 2007 cm^−1^). Thus, all of these signals can be associated with transitions involving these two vibrations. Two pronounced positive signals **9** and **10** at pump/probe frequencies of 1982/1959 and 2007/1993 cm^−1^ can be assigned to excited state absorption (**1-2**) of the two *ν*(tCO) modes (Fig. 2A). The difference between **0-1** and **1-2** transition frequencies yields anharmonicities of 23 and 14 cm^−1^ for the *ν*(tCO) modes at fundamental frequencies of 1982 and 2007 cm^−1^, respectively. While the first value is surprisingly high (*vide infra* for a detailed discussion), both values are lower than expected for bond-localized *ν*(CO) modes in this frequency regime (*ca.* 26 cm^−1^),^17^ indicating notable anharmonic coupling between the two vibrational degrees of freedom. Consistently, we observe off-diagonal (negative) bleach signals, **11** and **12**, at pump/probe frequencies of 1982/2008 and 2007/1983 cm^−1^ (Fig. 2A). Like all other cross peaks discussed in the following, both features are more pronounced in spectra recorded with perpendicular pump–probe polarization, as expected for signals arising from two vibrational modes with transition dipole moments at an angle above 45° (see Fig. S6† for a 2D-IR spectrum recorded with parallel pump–probe polarization). The first signal reflects a bleaching of the ground state of the higher-frequency *ν*(tCO)_2007_ mode upon pumping the lower-frequency *ν*(tCO)_1982_ mode (**00-01**), while the second signal reflects the opposite process (**00-10**). Observation of these signals demonstrates that the two *ν*(tCO) modes share a common vibrational ground state, *i.e.* they are anharmonically coupled. Furthermore, we detect an intense off-diagonal positive signal **13** at pump/probe frequencies of 2007/1970 cm^−1^ (Fig. 2A). This signal reflects the excitation of the *ν*(tCO)_1982_ mode starting from the first excited state of the *ν*(tCO)_2007_ mode (**01-11**). The difference between **00-10** and **01-11** probe frequencies is the intermode anharmonicity, which reflects the strength of the anharmonic coupling between the two vibrational degrees of freedom. We obtain a value of 13 cm^−1^, which indicates strong coupling between the two *ν*(tCO) modes. Based on this value, another positive signal **14** would be expected at pump/probe frequencies of 1982/1995 cm^−1^, reflecting excitation of the *ν*(tCO)_2007_ mode starting from the first excited state of the *ν*(tCO)_1982_ mode (**10-11**). While clear positive intensity exists between the negative bleach signals **7** and **11** at pump/probe frequencies of 1982/1982 and 1982/2007 cm^−1^ (Fig. 2A and S4†), the signal is not very well-defined and the position cannot be accurately determined. We assume that this is due to its inherently lower intensity as well as partial cancellation by the neighbouring bleach signals, especially the pronounced diagonal feature **7** at 1982/1982 cm^−1^. In addition to all previously mentioned features, we observe an unexpected signal **15** at 2007/1954 cm^−1^ (Fig. 2A). We tentatively assign this feature to a fifth-order transition that involves a total of three vibrational quanta, *e.g.* excitation of the *ν*(tCO)_1982_ mode starting from the second excited state of the *ν*(tCO)_2007_ mode (**02-12**). Besides the described dominant features, we notice two further faint positive *ν*(tCO) signals **16** and **17** at pump/probe frequencies of *ca.* 1982/2018 and 2007/1938 cm^−1^ (Fig. 2A, S4 and S5†), which can be assigned to dipole–forbidden transitions (**10-02** and **01-20**, respectively). Signals from dipole–forbidden transitions have also been observed for the *ν*(CN) modes of [NiFe] hydrogenases,^20^ and their observation confirms strong coupling between the two *ν*(tCO) modes of the H_inact_ state of *Dd*HydAB.

After analysing the three sets of vibrational reporters (*ν*(μCO), *ν*(tCO), and *ν*(tCN)) separately, we next turn to interactions between them. We detect off-diagonal signals **18** to **20** at pump/probe frequencies of 1847/1983 and 1982/1847 cm^−1^ (bleaching) as well as 1982/1846 cm^−1^ (excited-state absorption), see Fig. 2A and C. These cross peaks reveal apparent intermode anharmonicities below 1 cm^−1^, indicating very weak coupling between the *ν*(μCO)_1847_ mode and the *ν*(tCO)_1982_ mode. We also observe off-diagonal signals **21** to **24** at pump/probe frequencies of 1847/2007 and 2007/1847 cm^−1^ (bleaching) as well as 1847/2006 and 2007/1846 cm^−1^ (excited-state absorption), see Fig. 2A and C. These cross peaks reveal apparent intermode anharmonicities of 2 and 1 cm^−1^, indicating weak coupling between the *ν*(μCO)_1847_ mode and the *ν*(tCO)_2007_ mode. Weak interactions between each of the two *ν*(tCO) modes and the lower-frequency *ν*(tCN)_2087_ mode can also be observed. Specifically, we detect signals **25** to **28** at pump/probe frequencies of 2007/2087 and 2088/2007 cm^−1^ (bleaching) as well as 2007/2086 and 2088/2006 cm^−1^ (excited-state absorption), see Fig. 2B and E. The extracted anharmonicities are 1 and 2 cm^−1^, respectively. While these and similarly small values are difficult to determine accurately, the observation of the underlying signals indicates weak coupling between the *ν*(tCO)_2007_ mode and the *ν*(tCN)_2087_ mode. In addition, we notice faint cross peaks **29** to **32** at pump/probe frequencies of 1982/2087 and 2088/1982 (bleaching) as well as 1982/2090 and 2088/1985 (excited-state absorption), see Fig. 2E and F. These pairs of signals reveal a negative anharmonicity of *ca.* −3 cm^−1^, indicating weak interactions between the lower-frequency *ν*(tCO)_1982_ mode and the *ν*(tCN)_2087_ mode. Here, the negative sign can be interpreted as a strengthening of the *ν*(tCO) coordinate upon exciting the *ν*(tCN) mode, and *vice versa* (opposite to ‘normal’ anharmonic behaviour). No interactions between *ν*(μCO) and *ν*(tCN) modes can be detected. Moreover, the higher-frequency *ν*(tCN)_2107_ mode seems to be vibrationally isolated and not interacting with any of the other modes.

In summary, the described experimental observations demonstrate that the *ν*(μCO) and *ν*(tCN) modes represent stretching vibrations of individual μCO and tCN ligands. Thus, they can be utilized as highly localized (bond-specific) vibrational probes that can provide insights into, *e.g.*, the immediate protein environment of the associated ligands. The two *ν*(tCO) modes, on the other hand, represent a strongly coupled pair that cannot be interpreted in the same manner. In addition, both *ν*(tCO) modes also interact weakly with the *ν*(μCO) mode and the lower-frequency *ν*(tCN) mode.

Based on these observations, further analysis is necessary to extract structural information from the spectroscopic signature of the strongly coupled *ν*(tCO) modes. We have recently demonstrated that generalized second-order vibrational perturbation theory (GVPT2) is a powerful tool for understanding the structural information encoded in 2D-IR spectra of complex cyanido metal carbonyl compounds.^18^ Specifically, we found that intramode and intermode anharmonicities associated with the strongly coupled *ν*(CN) modes of [NiFe] hydrogenases yield valuable insights into the symmetry of the active site. Here, we use a similar approach to understand the pair of coupled *ν*(tCO) modes of [FeFe] hydrogenases. Again, we focus on the analysis of intramode and intermode anharmonicities for two major reasons: (1) if resonant interactions are properly taken into account (*vide infra*), anharmonicities yield direct insights into the shape of the potential energy surface along individual molecular coordinates, *e.g.* individual bonds, and the structural interactions between them. (2) Anharmonicities can be calculated with high accuracy since contributions from many error-prone (and method-dependent) quantities that spoil harmonic frequencies and absolute anharmonic transition energies cancel out in these relative quantities. We like to stress that anharmonic GVPT2 calculations are considerably more expensive than harmonic vibrational analyses but indispensable for understanding 2D-IR spectra since these do not exist within the harmonic limit.

**Fig. 4:**
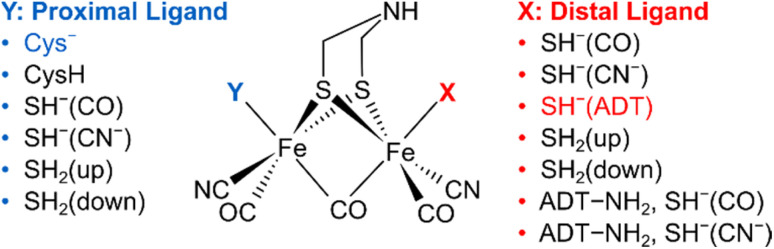
Schematic representation of computational models with variations of the apical ligands on the distal iron (*X*) and the proximal iron (*Y*). A protonated ADT bridging ligand was assumed for selected models, as indicated. The orientation of the apical ligands is qualitatively described. For details, see ESI (Fig. S7–S10 and Table S1).† The combination of ligands that yields the best fitting computational model #3 is highlighted.

**Fig. 5:**
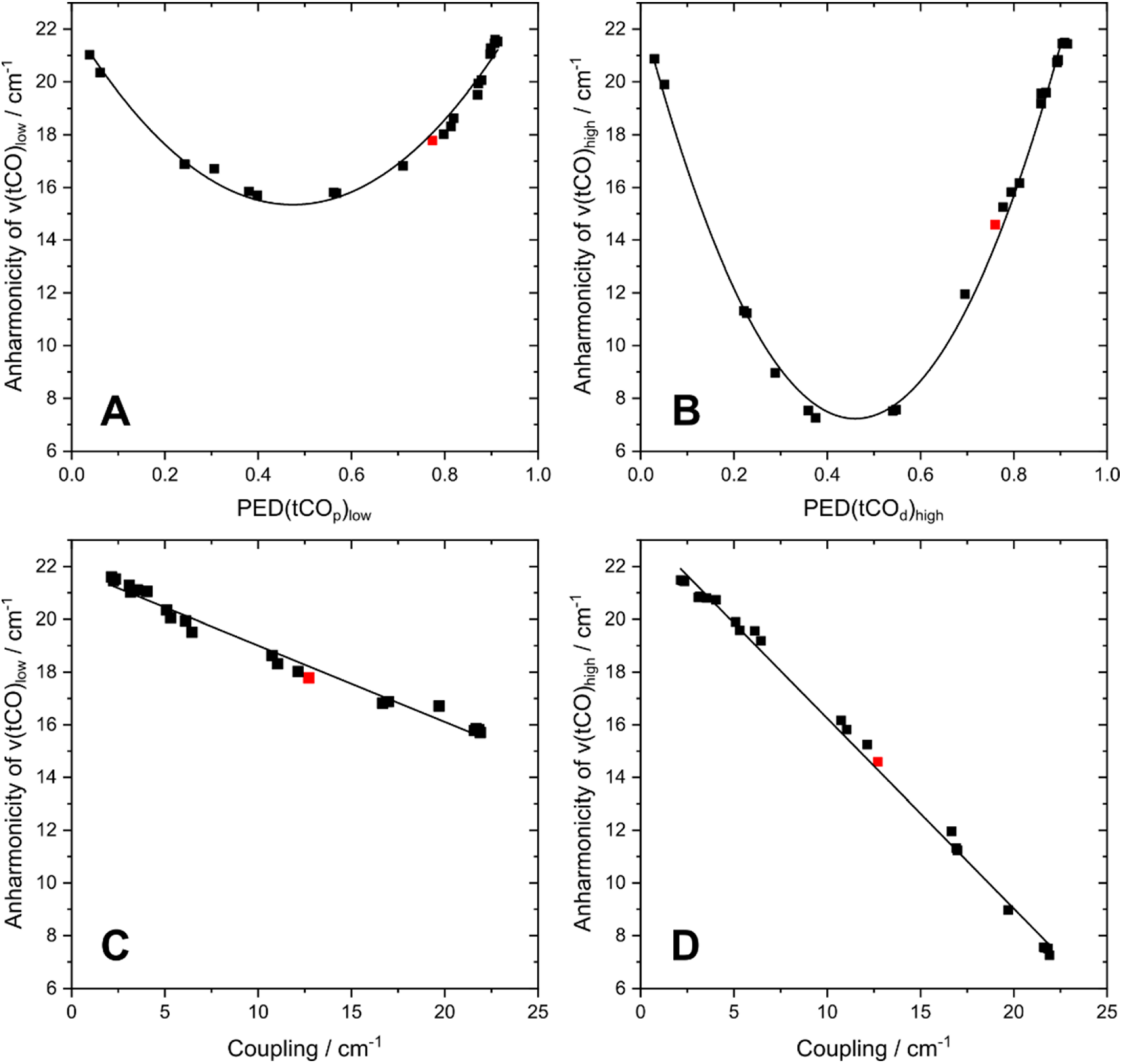
Interdependencies between computed anharmonicities and the potential energy distribution (PED) of terminal CO stretching modes, *ν*(tCO). (A) Dependence of the intramode anharmonicity of *ν*(tCO)_low_ on PED(tCO_p_)_low_. (B) Dependence of the intramode anharmonicity of *ν*(tCO)_high_ on PED(tCO_d_)_high_. (C) Interdependency between the intramode anharmonicity of *ν*(tCO)_low_ and the coupling (intermode anharmonicity) between the two *ν*(tCO) modes. (D) Interdependency between the intramode anharmonicity of *ν*(tCO)_high_ and the coupling (intermode anharmonicity) between the two *ν*(tCO) modes. *ν*(tCO)_low_ and *ν*(tCO)_high_ refer to the low-frequency and high-frequency *ν*(tCO) mode, respectively. PED(tCO_p_)_low_ and PED(tCO_d_)_high_ describe the localization of *ν*(tCO)_low_ and *ν*(tCO)_high_ on the proximal CO ligand, tCO_p_, and the distal CO ligand, tCO_d_, respectively. The degree of localization is quantified by the relative contributions of the associated bond stretching coordinates to the PED of the two modes. The datapoint corresponding to the best fitting computational model #3 is highlighted in red in all plots. For details, see Fig. S7–10.†

To understand the dependence of intramode and intermode anharmonicities on molecular structure we analysed 23 structural models of the [2Fe]_H_ subsite of the H-cluster in the H_inact_ state (Fig. 4, S7 and Table S1†). All of these models feature the consensus arrangement of CO and CN^−^ ligands, but they differ, in particular, in terms of the nature and/or relative orientation of the ligand bound to the axial positions of Fe_p_ and Fe_d_, respectively. We first analysed the intramode anharmonicities (Fig. S8†), reflecting the shape of the potential energy surface (PES) along the individual vibrational coordinates, in terms of their dependence on mode localization, as evaluated *via* the potential energy distribution (PED) of the normal modes (Fig. S9†). Specifically, we calculated bond-stretch contributions of the proximal tCO ligand (tCO_p_) and the distal tCO ligand (tCO_d_) to the potential energy of the low-frequency *ν*(tCO)_low_ mode and the high-frequency *ν*(tCO)_high_ mode, respectively. The dependence of the intramode anharmonicity on the derived mode localisation is illustrated for the *ν*(tCO)_low_ mode in Fig. 5A. Here, the abscissa shows the relative contribution of the tCO_p_ bond-stretching coordinate to the potential energy of this mode, PED(tCO_p_)_low_, while the ordinate reflects the intramode anharmonicity of this mode. In this representation, data points with PED(tCO_p_)_low_ values close to 1 reflect structural models for which the *ν*(tCO)_low_ mode is largely localized on tCO_p_. In other words, the vibrational mode is best described as a stretching vibration of the proximal CO ligand, *ν*(tCO_p_). By contrast, data points with PED(tCO_p_)_low_ values close to 0 reflect structural models for which the *ν*(tCO)_low_ mode is largely localized on the distal tCO ligand, tCO_d_, and best described as a stretching vibration of this CO ligand, *ν*(tCO_d_). Finally, PED(tCO_p_)_low_ values close to 0.5 reflect models where the *ν*(tCO)_low_ mode has nearly equal contributions from both tCO ligands and is best described as the antisymmetric stretching vibration of the two, *ν*(tCO)_asym_. Notably, a strong variation of PED(tCO_p_) values can be observed for the different structural models, including both delocalization and localization on either tCO_p_ or tCO_d_. Moreover, the intramode anharmonicity varies systematically with this quantity, yielding a parabolic dependence. This finding demonstrates that the intramode anharmonicity is maximized if the vibrational mode is localised on either of the two ligands and minimized in the delocalized limit. Similar behaviour is observed for the *ν*(tCO)_high_ mode (Fig. 5B), which can be assigned to the symmetric stretching vibration, *ν*(tCO)_sym_, in the delocalized limit. Finally, we notice that the parabolic dependence of the intramode anharmonicity on mode localization is more pronounced for the *ν*(tCO)_high_ mode than for the *ν*(tCO)_low_ mode (*cf.*Fig. 5A and B). The difference in intramode anharmonicities is most pronounced in the delocalized limit, PED(tCO_d/p_)_low/high_ ≈ 0.5, which corresponds to strong coupling between the two modes (*vide infra*). This difference can be largely explained by a 2-2 Darling–Dennison (DD) resonance that lowers the energy of the second excited state of the lower-frequency *ν*(tCO) mode and raises the energy of the second excited state of the higher-frequency *ν*(tCO) mode.^18^

We next inspect the intermode anharmonicities, which exhibit a trend opposite to that of the intramode anharmonicities, for both *ν*(tCO) modes. This is most easily seen by a negative linear relation between the intramode and intermode anharmonicities (Fig. 5C and D). The observed behaviour can be explained by the dependence of intermode coupling on spatial mode overlap and, thus, mode delocalization. In the localized limit, the normal mode vectors of *ν*(tCO_p_) and *ν*(tCO_d_) have zero spatial overlap (different ligands vibrate in each case), so that the coupling is weak and the intermode anharmonicity small. In the delocalized limit, *ν*(tCO)_asym_ and *ν*(tCO)_sym_ have maximum spatial overlap (both ligands vibrate in both cases), so that the coupling is strong and the intermode anharmonicity large. While the qualitative trend is identical for both *ν*(tCO) modes, we note differences in the slope of the linear function that describes the interplay of intramode and intermode anharmonicities. Again, this difference can be explained by resonant splitting of second excited states (*vide supra*), which affects the intramode anharmonicities (but not the intermode anharmonicities). This resonant splitting needs to be considered when deriving information about the shape of the PES along the individual vibrational coordinates.

In summary, the described analysis demonstrates that the intramode and intermode anharmonicities can be directly related to the nature of the probed normal modes, which may be described as either bond-localized stretching vibrations or (anti)symmetric combinations in the two limiting cases. This observation is relevant for two reasons: (1) mode localization is directly related to molecular symmetry, *i.e.* differences in the two CO ligands lead to localized modes. The degree of asymmetry in molecular and electronic structure, as also observed in [FeFe] model complexes,^39^ can therefore be quantified through increasing intramode anharmonicities and decreasing intermode anharmonicities. (2) Evaluation of mode localization *via* these anharmonicities provides a quantitative description of the normal mode vectors (atom displacements), which can be easily computed by standard harmonic vibrational analyses. While normal mode eigenvalues (squared vibrational frequencies) are routinely used to validate computational models of molecular structure, normal mode eigenvectors are typically left unused as they are difficult to extract from linear IR absorption experiments. The outlined analysis introduces anharmonicities as sensitive and easily accessible observables that can be utilized to quantitatively validate structural models by making full use of the information available from computational vibrational analyses.

Based on the introduced analysis, we next aimed to identify the best fit among the tested structural models. Large intramode anharmonicities of *ν*(μCO) and *ν*(tCN) modes, reflecting localization of these modes to individual ligand bond coordinates, are well reproduced in all structural models. This observation has two important implications: (1) the localization of these modes, as observed in the experiment, is not a consequence of the protein environment, as evident from their robust reproduction in first-coordination-sphere computational models. (2) Anharmonicities of *ν*(μCO) and *ν*(tCN) modes are unsuited for analysing the structural variations reflected by the H-cluster models under investigation. We like to stress, though, that these observables might still yield valuable information regarding other structural aspects, *e.g.* variations in (H-bonding) interactions with the protein environment. Moreover, we find that the site of mode localization differs across the individual structural models. In most cases, the lower-frequency *ν*(tCN) mode is localized on the proximal tCN ligand, tCN_p_, while the higher-frequency *ν*(tCN) mode is localized on the distal tCN ligand, tCN_d_. In rare cases, however, mode localization is inverted. This observation fits to previous reports indicating that *ν*(tCN) mode localization is not conserved across structural states and [FeFe] hydrogenases from different organisms and, thus, a sensitive structural marker.^38^

**Fig. 6:**
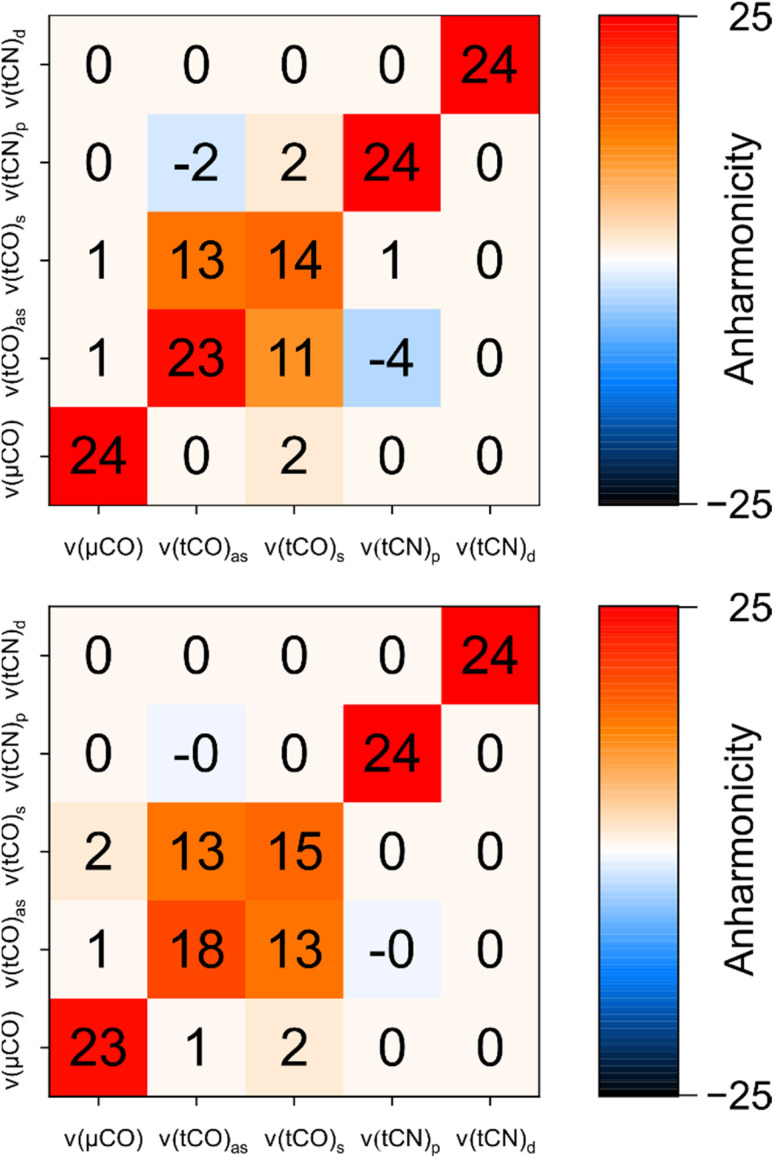
Comparison of experimental anharmonicities (top) and anharmonicities computed for the best fitting structural model of H_inact_ (model #3; bottom). All values are given in units of cm^−1^ and rounded to the nearest integer. Values flagged as −0 indicate negative values with a magnitude <0.5. For details, see Fig. S7–S10 and Table S1.†

In contrast to intramode anharmonicities of *ν*(μCO) and *ν*(tCN) modes, both intramode and intermode anharmonicities of *ν*(tCO) modes vary strongly with molecular structure (*vide supra*). Thus, these observables represent valuable reporters for changes in the first coordination sphere of the [2Fe]_H_ subsite of the H-cluster. We find that most of our models fall into one of two categories: the first one exhibits large and equal intramode anharmonicities for the two modes as well as a small intermode anharmonicity. This behaviour represents the localized limit with two weakly coupled single-bond stretch modes. The second group exhibits a moderately large intramode anharmonicity for the lower-frequency *ν*(tCO) mode and a small one for the higher-frequency *ν*(tCO) mode. Moreover, the intermode anharmonicity is large, reflecting the delocalized limit with strongly coupled (and resonantly interacting) *ν*(tCO)_asym_ and *ν*(tCO)_sym_ modes. The experimental data lie between these two limiting cases, *i.e.* the two intramode anharmonicities exhibit slightly different, moderate values, and also the intermode anharmonicity lies about halfway between the two extreme cases. This indicates partly localized (tCO)_asym_ and *ν*(tCO)_sym_ modes resulting from two interacting and slightly different CO oscillators. Only a limited number of computational models approximate this behaviour. The best fitting model #3 (Fig. 1, 4, 6 and S7–S10†) contains an SH^−^ ligand on Fe_d_, pointing towards the ADT bridgehead, and a deprotonated cysteine coordinated to Fe_p_. This scenario fits to the proposed structure for the H_inact_ state,^13^ supporting the reliability of the introduced analysis and the previously suggested structure. In addition, the utilized analysis provides the first indication for an interaction of the HS^−^ ligand with the ADT bridgehead. Finally, inspection of PED values of the best-fitting model indicates that the two *ν*(tCO) modes are best described as *ν*(tCO)_asym_ and *ν*(tCO)_sym_, partly localized on tCO_p_ and tCO_d_, respectively. We conclude that the two CO ligands feature similar bond properties, indicating a quasi-symmetric charge distribution across Fe_d_ and Fe_p_, as expected for the di-ferrous ground state of H_inact_.

While the intermode anharmonicity and the intramode anharmonicity of the *ν*(tCO)_2007_ mode are reproduced within 1 cm^−1^ or better, a somewhat larger deviation is observed for the intramode anharmonicity of the *ν*(tCO)_1982_ mode (Fig. 6). While limitations in the accuracy of the calculations cannot be excluded *per se*, we like to stress that the computed value is reasonable, while the experimental value appears surprisingly high (*vide supra*). Specifically, the experimentally observed intramode anharmonicity of 23 cm^−1^ is close to the localized limit, which is incompatible with the moderate intramode anharmonicity of the higher-frequency *ν*(tCO) mode (14 cm^−1^) and the non-negligible coupling between the two (13 cm^−1^). Since the difference in intramode anharmonicities of the two *ν*(tCO) modes results from resonant interactions in the first place (*vide supra*), we propose that the unusually high intramode anharmonicity of the *ν*(tCO)_1982_ mode also results from a resonant interaction, specifically one that affects this mode but not the *ν*(tCO)_2007_ mode. A reasonable candidate would be 1-3 DD resonance involving the first excited state of the lower-frequency *ν*(tCO)_1982_ mode and a triple excitation of low-frequency modes, *e.g.* the second overtone of a mode related to metal–ligand stretching or bending.^40^ Such an interaction, which would be difficult to capture in calculations, could selectively increase the energy of the first excited state of the *ν*(tCO)_1982_ mode, thereby increasing its intramode anharmonicity (Fig. S11†). While such a phenomenon should also have some effect on the intermode anharmonicity, the effect on the intramode anharmonicity would be twice as large.

As illustrated above, intramode and intermode anharmonicities associated with the *ν*(tCO) modes of [FeFe] hydrogenases are sensitive experimental observables that report on the [2Fe] core structure of the H-cluster. In the following, we will briefly discuss this observation based on differences between *ν*(tCO) and *ν*(tCN) modes in both [FeFe] and [NiFe] hydrogenases. The statements made in the following are based on insights from the current study, previous work on [NiFe] hydrogenases,^4,12,17–20^ and general aspects of CO/CN^−^ bonding in transition metal compounds. Further work, *e.g.* on other [FeFe] and [NiFe] states, might be necessary to confirm and expand these interpretations.

For [FeFe] hydrogenases, *ν*(tCO) modes are coupled and partly delocalized across the two terminal CO ligands. *ν*(tCN) modes, on the other hand, are bond-localized and uncoupled in these enzymes but delocalized and strongly coupled in [NiFe] hydrogenases. All these observations are reproduced by GVPT2 calculations on first-coordination-sphere models, indicating that these aspects are fundamentally dictated by the electronic core structures of [FeFe] and [NiFe] active sites. We propose that the observed behaviours can be explained by differences in metal-binding by CO and CN^−^ ligands as follows.

CO is generally accepted to bind to low-valent transition metal centres, mainly, by means of strong π backbonding. CN^−^, on the other hand, is a strong σ donor with only moderate backbonding capabilities. Due to its negative net charge, CN^−^ can also interact with transition metal ions electrostatically, and this capability has been suggested to dominate over orbital interactions.^41^ As a consequence, CO coordination is largely controlled by the occupancy of metal t_2g_ orbitals, while CN^−^ coordination is governed by e_g_ orbital occupancy and the net charge of the coordinated metal ion. Of note, vibrationally induced modulations of these properties will mediate interactions between CO and CN^−^ ligand oscillators, thereby leading to the potential formation of delocalized and coupled vibrational modes. Within this framework, the delocalization and coupling of *ν*(tCO) modes of [FeFe] hydrogenases can be explained by strong interactions between the two terminal CO ligands *via* the electron density related to the fully occupied t_2g_ levels of the two low-spin ferrous iron centres. Interactions between the two CN^−^ ligands of [FeFe] hydrogenases are much weaker since e_g_ levels are unoccupied in low-spin ferrous iron, therefore leading to bond-localized and uncoupled *ν*(tCN) vibrations. The stronger coupling and pronounced delocalization of *ν*(tCN) modes in [NiFe] hydrogenases can be explained by the fact that the two CN^−^ ligands are bound to the same central metal ion in these enzymes. Under these conditions, interactions between the two CN^−^ ligands are stronger for three reasons: (1) both ligands are bound to the same e_g_ orbital, which facilitates direct crosstalk *via* bonding interactions. (2) For the same reason, electrostatic interactions mediated by the central metal ion will be stronger. (3) CN^−^ exhibits a considerable quadrupole moment,^41^ so that spatially close CN^−^ ligands may electrostatically interact through space.

We conclude that terminal CO ligands of [FeFe] hydrogenases interact *via* π backbonding and the modulation of electron density related to t_2g_ levels of the two iron ions. This leads to partly delocalized and coupled modes, and the level of delocalization, as probed by 2D-IR spectroscopy, allows insights into molecular structure and symmetry. Terminal CN^−^ ligands interact more weakly if spatially separated. Thus, they can be utilized as localized structural probes in [FeFe] hydrogenases where they are bound to two different metal ions. If bound to the same metal ion, as in [NiFe] hydrogenases, interactions between CN^−^ ligands become stronger, leading to delocalized and coupled modes that can be analysed again in terms of overall structure and symmetry.

## Conclusions and outlook

Here we have demonstrated how the combination of experimental and computational 2D-IR spectroscopy can be used to explore the vibrational and molecular structure of [FeFe] hydrogenases. We expect this approach to considerably expand the rich methodological toolbox available for studying these enzymes, thereby helping to answer open questions in the field, especially if combined with other spectroscopic methods in a synergistic manner.

Our findings show that intramode and intermode anharmonicities can be utilized to quantitatively describe the nature of the widely used CO and CN stretching reporter vibrations and their interactions, thereby gaining insights into the bonding and non-bonding crosstalk between the underlying ligands. In particular, the localization of vibrational modes associated with the stretching of the terminal CO ligands is found to be highly sensitive to the core structure and symmetry of the [2Fe]_H_ moiety of the H-cluster. Analysing this mode localization through computational models and experimentally derived anharmonicities, we find that the structure of the H_inact_ state is best described as containing an SH^−^ ligand bound to the distal iron and pointing towards the azadithiolate ligand. This model supports and extends previous proposals from crystallographic and computational studies,^13^ demonstrating the power of the introduced approach. In addition – and in contrast to X-ray diffraction experiments – all information is obtained in solution phase, under ambient conditions, and in a non-destructive manner. We therefore expect this strategy to gain unambiguous and biologically representative insights into the structure of other poorly explored or controversially discussed H-cluster intermediates.

Besides addressing pending questions in hydrogenase research, future work will focus on methodological challenges associated with the employed experimental and computational strategies. From the experimental perspective, this work will focus on strategies for dealing with complex mixtures of states. While 2D-IR spectroscopy yields a plethora of additional observables for structural interrogation, this benefit comes at the price of numerous signals that might overlap and cancel in typical mixtures of states observed for hydrogenases, *e.g.* under catalytic conditions. To address this challenge, future work might involve experiments with increased spectral resolution as well as polarization schemes that allow selecting individual Liouville pathways. The latter approach suppresses specific signals (*e.g.* from diagonal transitions),^42^ thereby facilitating their identification and the analysis of the remaining spectral features. In addition, the interpretation of strongly overlapping spectral contributions will benefit from the advancement of computational protocols to yield a truly predictive approach. Our current strategy has focussed on first-coordination sphere models of the [2Fe] centre for two reasons. First, GVPT2 scales unfavourably with the number of atoms *N*, since up to 6*N* − 11 Hessian matrices have to be calculated. Second, the perturbative approach is unsuitable for the treatment of large-amplitude low-frequency modes, the number of which increases with the size of the models. To deal with these challenges, we are currently exploring systematic schemes for performing GVPT2 calculations in reduced normal mode spaces, thereby reducing the number of anharmonically treated degrees of freedom, so that larger computational models can be analysed. These larger models will include the immediate protein environment and the covalently linked [4Fe–4S] cluster, both of which might have an impact on the electronic structure and geometry of the H-cluster and, thus, the vibrationally probed asymmetry in charge distribution. In addition, this approach might help to predict resonances involving high-frequency and low-frequency modes with sufficient accuracy. We expect these advances to further strengthen the illustrated approach of combining experimental and computational 2D-IR spectroscopy for studying [FeFe] hydrogenases. In a more general sense, the current study highlights the potential of this approach for studying complex, polynuclear organometallic targets in biology and beyond.

## Data availability

Data supporting this article are included in the main text and the ESI.† Additional data are available from the authors upon reasonable request.

## Author contributions

**Cornelius C. M. Bernitzky**: data curation, formal analysis, investigation, software, validation, visualization, writing – original draft, writing – review & editing; **Yvonne Rippers**: data curation, formal analysis, investigation, methodology, software, validation, visualization, writing – review & editing; **Denise Poire**: formal analysis, investigation, writing – review & editing; **Mathesh Vaithiyanathan**: formal analysis, investigation, writing – review & editing; **Solomon L. D. Wrathall**: investigation, writing – review & editing; **Barbara Procacci**: investigation, writing – review & editing; **Igor V. Sazanovich**: methodology, writing – review & editing; **Gregory M. Greetham**: methodology, software, writing – review & editing; **Patricia Rodríguez-Macía**: funding acquisition, resources, writing – review & editing; **Neil T. Hunt**: funding acquisition, supervision, writing – review & editing; **James Birrell**: funding acquisition, investigation, resources, validation, writing – review & editing; **Marius Horch**: conceptualization, data curation, formal analysis, funding acquisition, investigation, methodology, project administration, supervision, validation, visualization, writing – original draft, writing – review & editing.

## Conflicts of interest

There are no conflicts to declare.

## Supplementary Material

SC-OLF-D5SC01811K-s001
