# Supplementary material for: Two-dimensional infrared spectroscopy as a tool to reveal the vibrational and molecular structure of [FeFe] hydrogenases
Source: Chem Sci. 2025 May 7;16(24):10957–69. doi: 10.1039/d5sc01811k (PMC12086866; doi:10.1039/d5sc01811k)
Supplement: SC-016-D5SC01811K-s001 [file SC-016-D5SC01811K-s001.pdf]

Supplementary Information (SI) for Chemical Science.
This journal is © The Royal Society of Chemistry 2025

Electronic Supplementary Information

Two-dimensional Infrared Spectroscopy as a Tool to Reveal the Vibrational and Molecular Structure of [FeFe] Hydrogenases

Cornelius C. M. Bernitzky,[‡a] Yvonne Rippers,[‡a] Denise Poire,[a,b] Mathesh Vaithiyanathan,[a] Solomon L. D. Wrathall,[c] Barbara Procacci,[c] Igor V. Sazanovich,[d] Gregory M. Greetham,[d] Patricia Rodríguez-Macía,[e] Neil T. Hunt,[c] James A. Birrell,[f] Marius Horch*[a]

[a] Freie Universität Berlin, Department of Physics, Ultrafast Dynamics in Catalysis, Arnimallee 14, 14195 Berlin, Germany. E-mail: marius.horch@fu-berlin.de

[b] Technische Universität Berlin, Department of Chemistry, Modeling of Biomolecular Systems, Straße des 17. Juni 135, 10623 Berlin, Germany

[c] Department of Chemistry and York Biomedical Research Institute, University of York, York, YO10 5DD, UK

[d] STFC Central Laser Facility, Research Complex at Harwell, Rutherford Appleton Laboratory, Harwell Campus, Didcot, OX11 0QX, UK

[e] School of Chemistry, University of Leicester, University Rd, Leicester, LE1 7RH, UK

[f] School of Life Sciences, University of Essex, Wivenhoe Park, Colchester, CO4 3SQ, UK

[‡] These authors contributed equally. Their names are listed alphabetically.

Contents

Supplementary Figures S1–S11 p. S1

Supplementary Table S1 p. S12

Atomic Coordinates of Computational Models p. S13

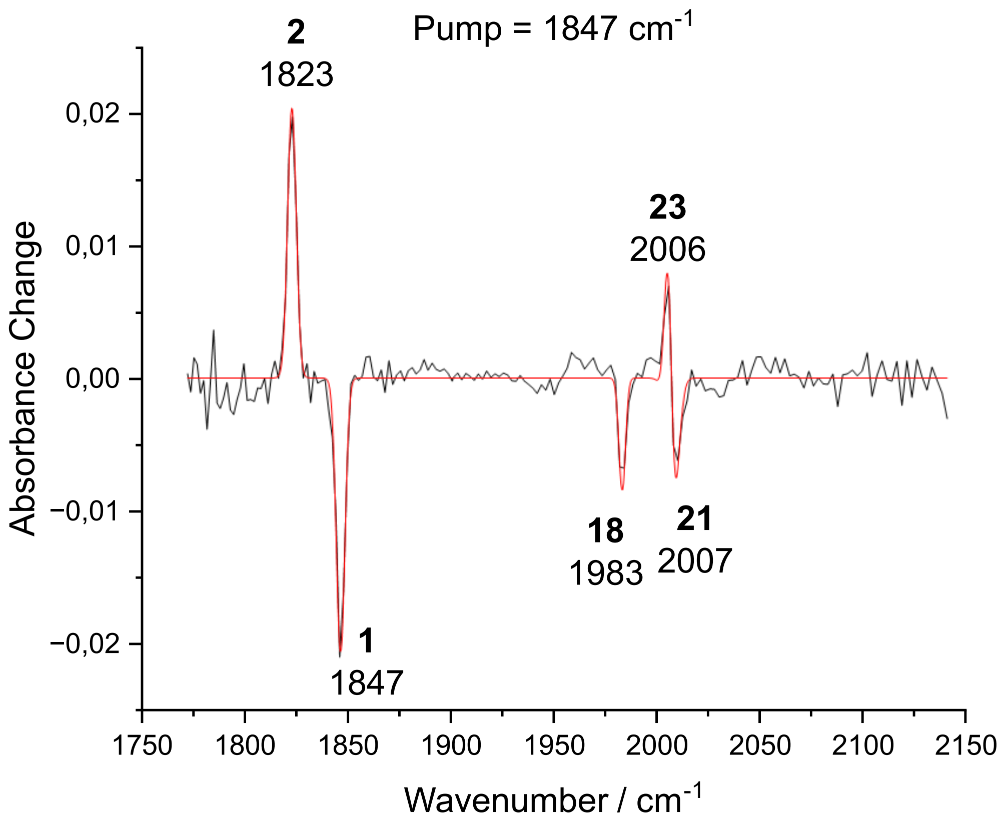

Figure S1: Pump slice (1847 cm^{-1}) of a 2D-IR spectrum of *Dd*HydAB enriched in the H_{inact} state (black trace), obtained at a waiting time of T_w = 250 fs (see Fig. 2 of the manuscript). The spectrum was recorded with perpendicular polarization of pump and probe pulses. The red trace represents the sum of five Gaussian lineshape functions fitted to the experimental spectrum.

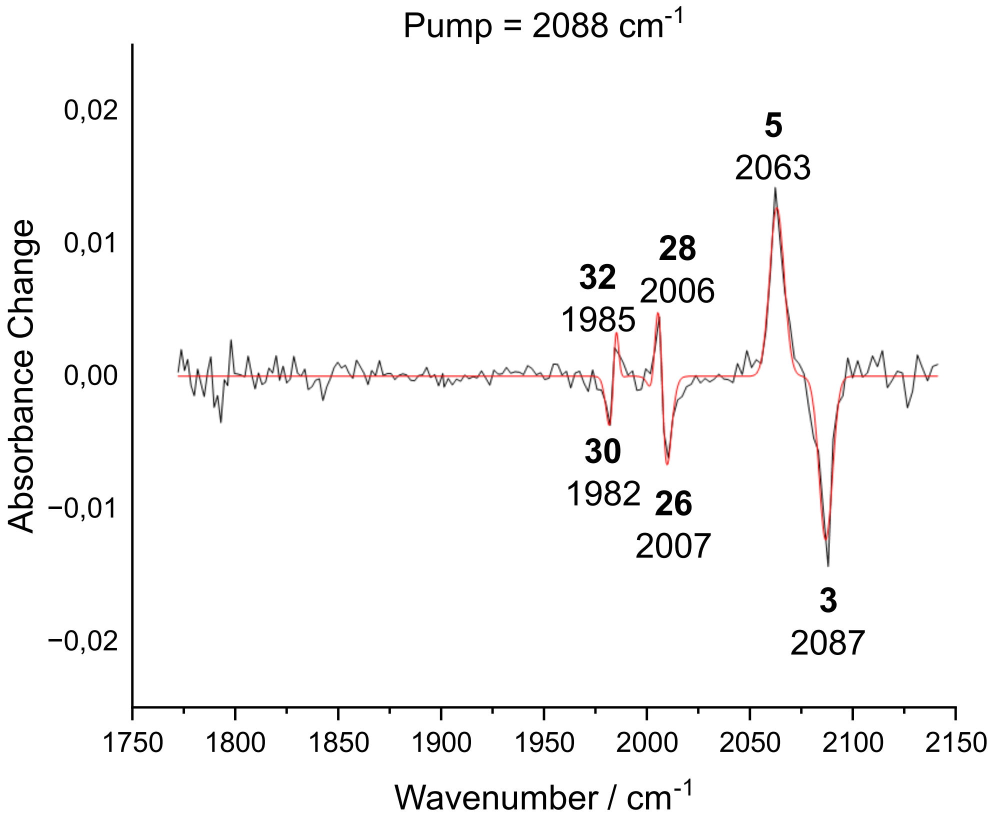

Figure S2: Pump slice (2088 cm^{-1}) of a 2D-IR spectrum of *Dd*HydAB enriched in the H_{inact} state (black trace), obtained at a waiting time of T_w = 250 fs (see Fig. 2 of the manuscript). The spectrum was recorded with perpendicular polarization of pump and probe pulses. The red trace represents the sum of six Gaussian lineshape functions fitted to the experimental spectrum.

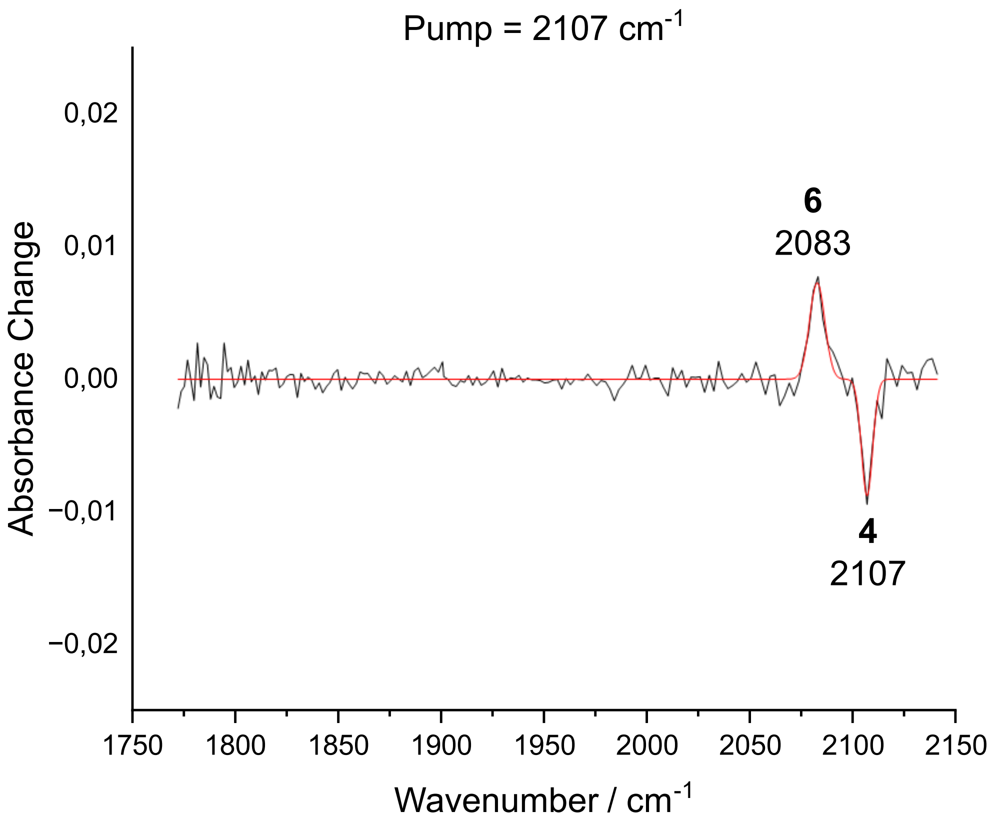

Figure S3: Pump slice (2107 cm^{-1}) of a 2D-IR spectrum of *Dd*HydAB enriched in the H_{inact} state (black trace), obtained at a waiting time of T_w = 250 fs (see Fig. 2 of the manuscript). The spectrum was recorded with perpendicular polarization of pump and probe pulses. The red trace represents the sum of two Gaussian lineshape functions fitted to the experimental spectrum.

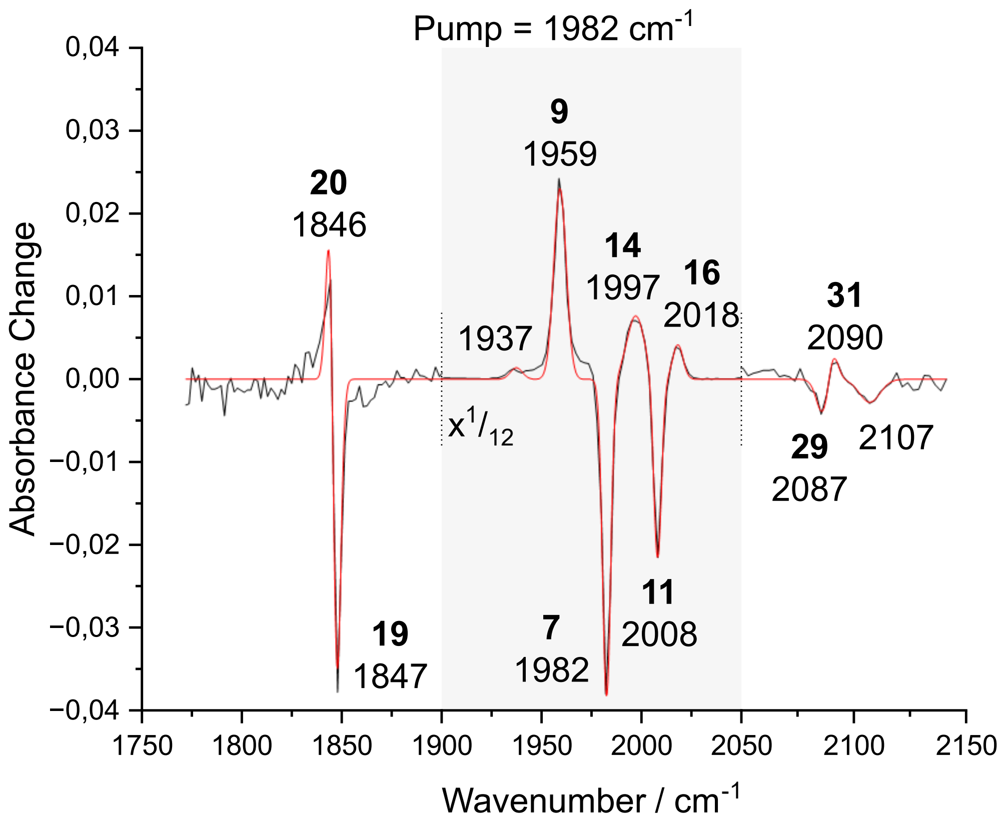

Figure S4: Pump slice (1982 cm^{-1}) of a 2D-IR spectrum of *Dd*HydAB enriched in the H_{inact} state (black trace), obtained at a waiting time of T_w = 250 fs (see Fig. 2 of the manuscript). The spectrum was recorded with perpendicular polarization of pump and probe pulses. The red trace represents the sum of eleven Gaussian lineshape functions fitted to the experimental spectrum. The grey region between 1900 and 2050 cm^{-1} is scaled down by a factor of 12 relative to the rest of the spectrum to keep weak signals visible. The weak signal at 1937 cm^{-1} could originate from 2-3 transition of the 1982 cm^{-1} mode (**7**). The origin of the signal at 2107 cm^{-1} is currently unclear.

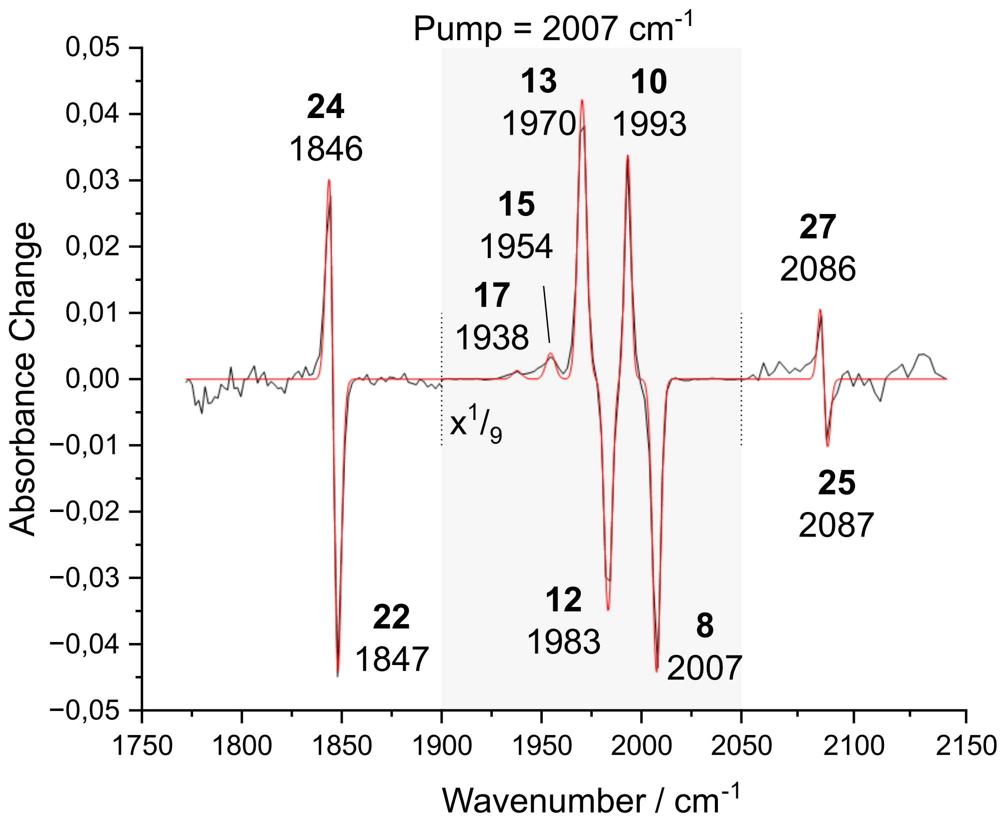

Figure S5: Pump slice (2007 cm^{-1}) of a 2D-IR spectrum of *Dd*HydAB enriched in the H_{inact} state(black trace), obtained at a waiting times of T_w = 250 fs (see Fig. 2 of the manuscript). The spectrum was recorded with perpendicular polarization of pump and probe pulses. The red trace represents the sum of ten Gaussian lineshape functions fitted to the experimental spectrum. The grey region between 1900 and 2050 cm^{-1} is scaled down by a factor of 9 relative to the rest of the spectrum to keep weak signals visible.

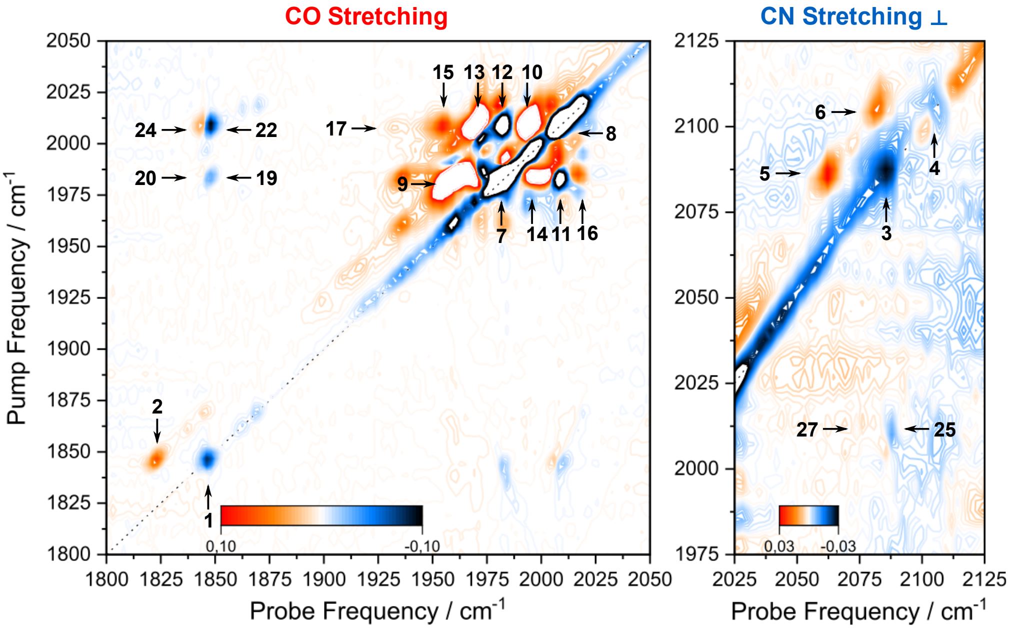

Figure S6: 2D-IR spectrum of *Dd*HydAB enriched in the H_{inact} state, obtained at a waiting time of T_w = 250 fs. Spectra were recorded with parallel polarization of pump and probe pulses.

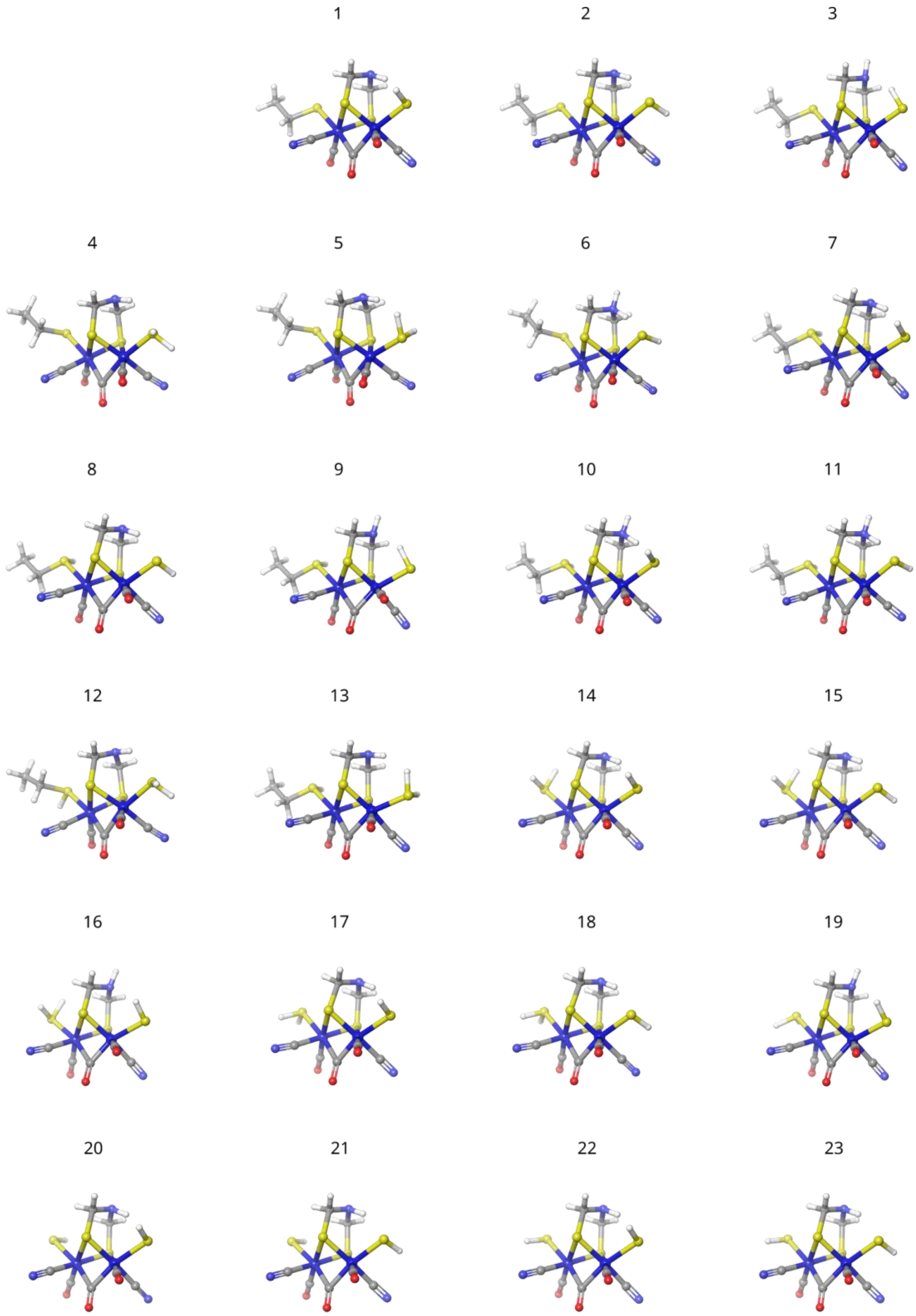

Figure S7: Structural depictions of all computational models, as detailed in Table S1. dark blue = iron, light blue = nitrogen, grey = carbon, red = oxygen, white = hydrogen, yellow = sulfur.

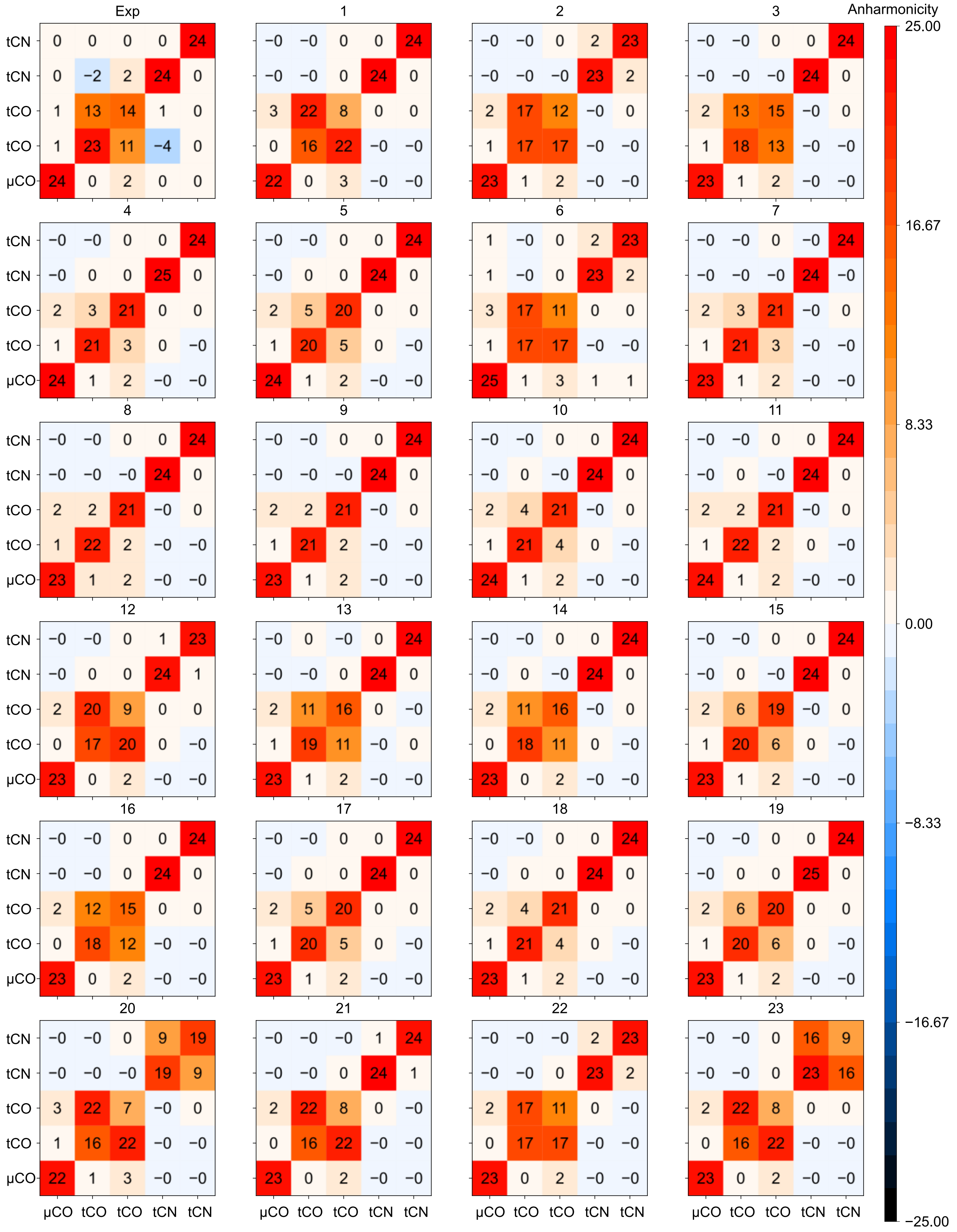

Figure S8: Matrix representation of diagonal and off-diagonal anharmonicities, as obtained in the experiment and for the indicated computational models (also see Table S1). All values are given in units of cm^{-1} and rounded to the nearest integer. Values flagged as −0 indicate negative values with a magnitude < 0.5.

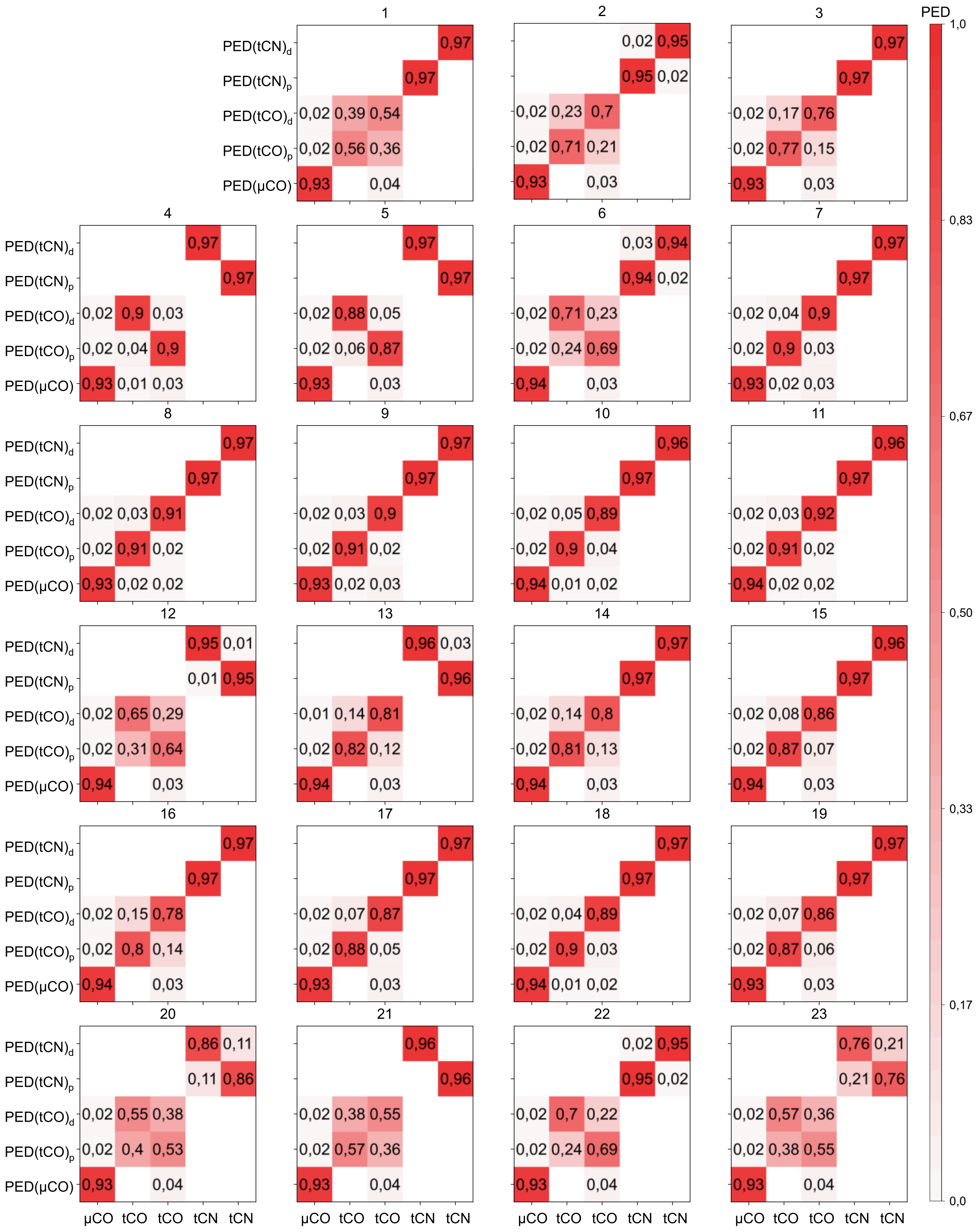

Figure S9: Matrix representation of PED contributions to the CX stretch vibrational modes (assuming values between 0 and 1), as obtained for the indicated computational models (also see Table S1). Only contributions ≥ 0.01 (1 %) are indicated.

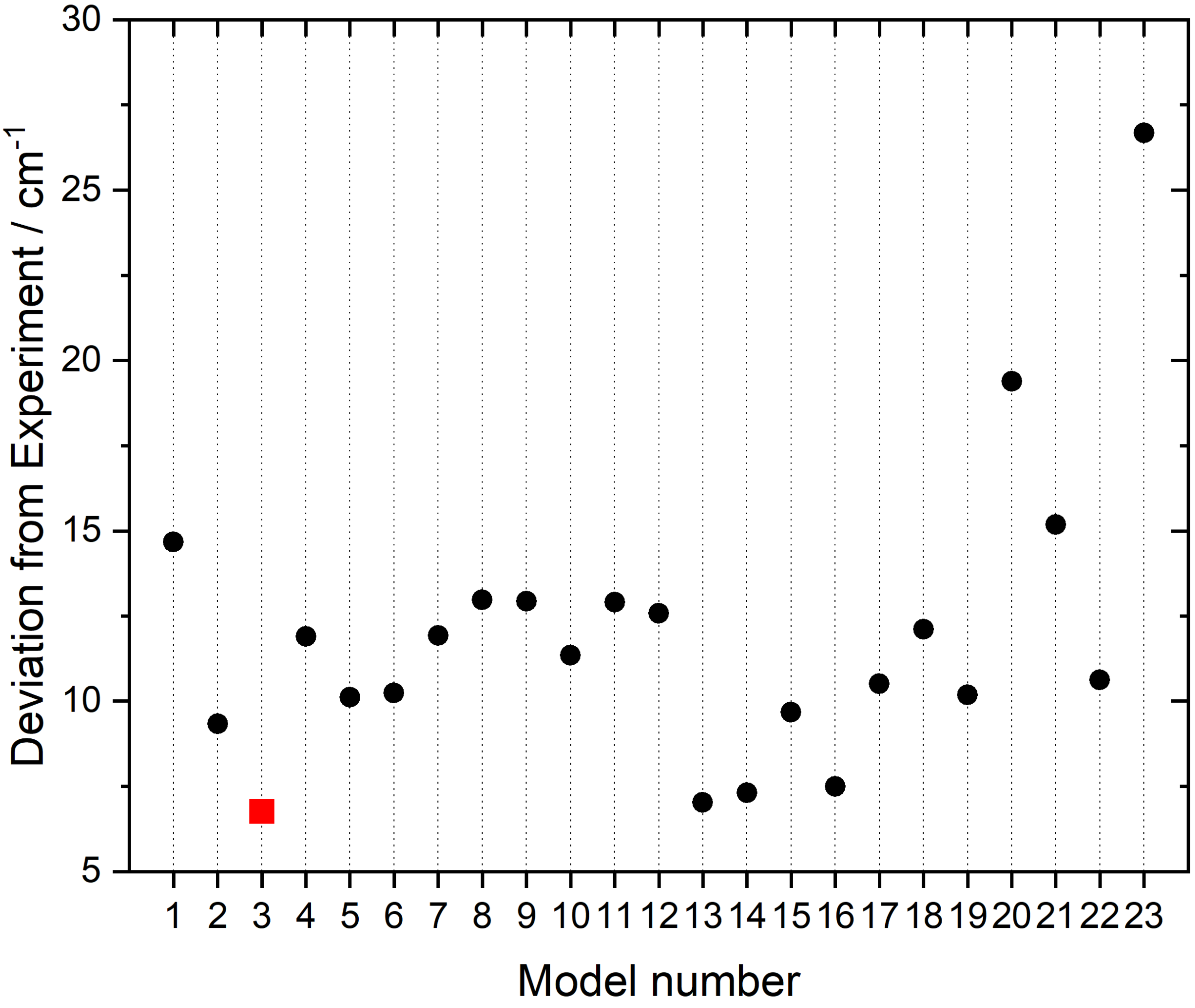

Figure S10: Overall deviation of calculated anharmonicities from the experimental values, plotted for all computational models. The deviation has been calculated as the Euclidean distance d between computed data and experimental data, both treated as points in the 15-dimensional space of diagonal and off-diagonal CO/CN stretch anharmonicities: $d = \sqrt{\sum_{i=1}^{5}\sum_{j\geq i}^{5}\Delta\Delta_{ij}{}^{2}}$. Here, $\Delta\Delta_{ij}$is the difference between the experimental and calculated anharmonicity associated with normal modes i and j, both of which refer to one of the five CO/CN stretching modes. For diagonal anharmonicities $i = j$, while for off-diagonal anharmonicities $i \neq j$. Note that for every pair of modes i and j, $\Delta_{ij}= \Delta_{ji}$. The formula given above accounts for this relation and avoids double counting of off-diagonal anharmonicities. In the experiment, apparent values of Δ_{ij} and Δ_{ji} are not exactly identical, though, due to differences in signal-to-noise ratio between cross peaks above and below the diagonal. For comparison with calculated values, the arithmetic mean of experimental Δ_{ij} and Δ_{ji} values was used to calculate $\Delta\Delta_{ij}$. The best fitting model #3 is highlighted in red.

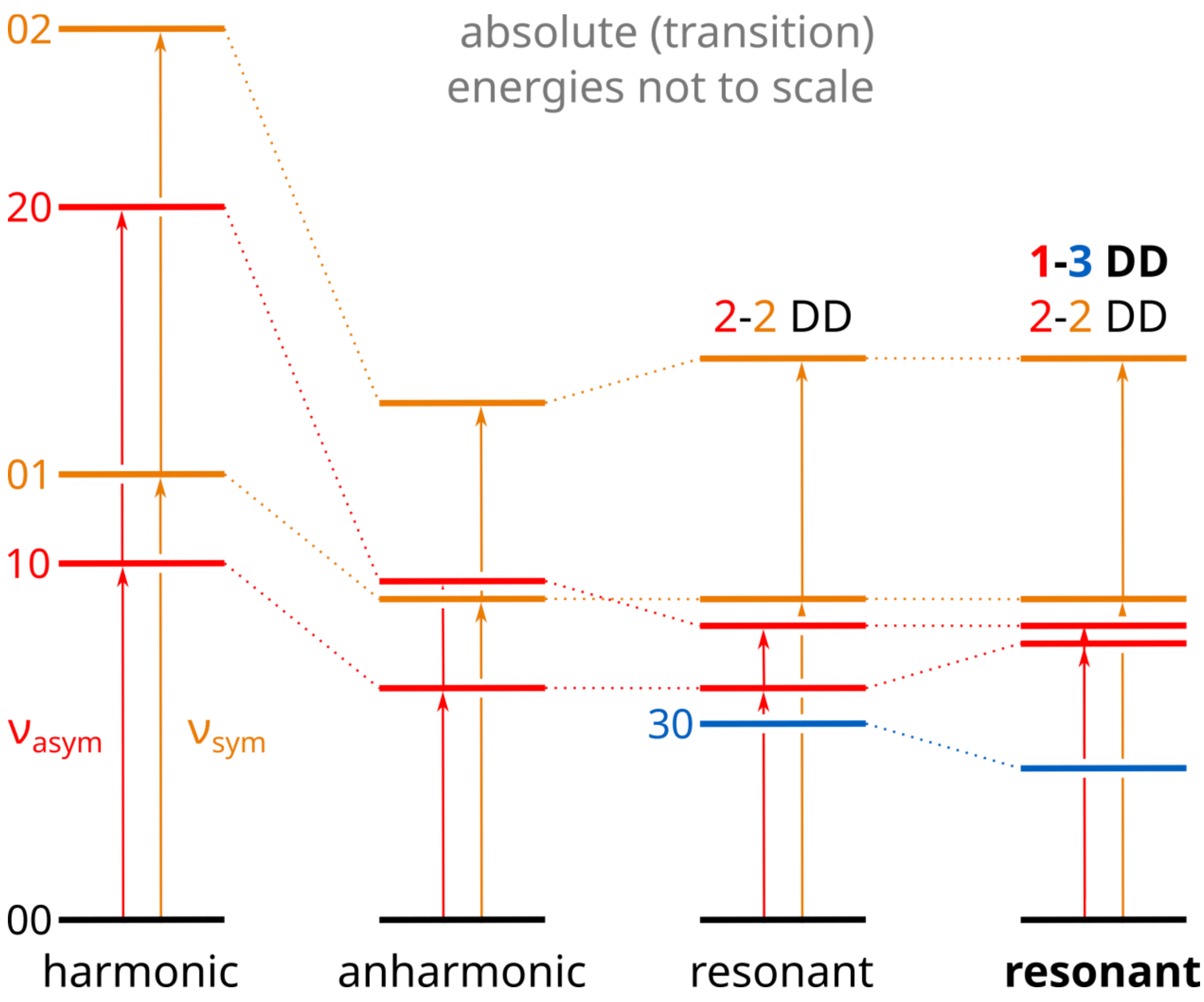

Figure S11: Energy level scheme for two interacting vibrational modes, e.g. symmetric and antisymmetric stretching modes (ν_{sym} and ν_{asym}). The impact of anharmonicity and different types of resonant interactions is illustrated. Anharmonicity leads to a lowering of all energy levels for both modes. A resonant interaction of the second excited states (**2**) of the two modes (**2-2** Darling-Dennison resonance) furthermore leads to a splitting of these energy levels. In addition, the first excited state (**1**) of the lower-frequency mode (antisymmetric stretching) resonantly interacts with a triple excitation, e.g. the second overtone of a low-frequency that is not directly detectable (**1-3** Darling-Dennison resonance). The latter phenomenon leads to a selective increase of the energy associated with first excited state of the antisymmetric stretch mode and, thus, the apparent anharmonicity (difference between **0-1** and **1-2** transition energies).

Table S1: Overview of computational models utilized in this study.‡

#	Ligand Fe_p	Ligand Fe_d	Bridge	Q	$\omega^h_{\mu CO}$	ω^h_{tCO}	ω^h_{tCO}	ω^h_{tCN}	ω^h_{tCN}	$\omega^a_{\mu CO}$	ω^a_{tCO}	ω^a_{tCO}	ω^a_{tCN}	ω^a_{tCN}
1	$Cys382^-$	SH^- (CO)	ADT-H	−2	1841	1949	1962	2104	2115	1818	1919	1932	2071	2091
2	$Cys382^-$	SH^- (CN^-)	ADT-H	−2	1839	1950	1964	2104	2109	1819	1919	1935	2079	2080
3	$Cys382^-$	SH^- (ADT)	ADT-H	−2	1840	1948	1964	2104	2115	1814	1920	1937	2076	2089
4	$Cys382^-$	SH_2 (down)	ADT-H	−1	1888	1974	2005	2113	2127	1856	1945	1974	2085	2100
5	$Cys382^-$	SH_2 (up, CN^-)	ADT-H	−1	1889	1982	2006	2115	2127	1862	1948	1973	2084	2096
6	$Cys382^-$	SH^- (CN^-)	ADT-H_2^+	−1	1878	1991	2004	2124	2127	1857	1964	1977	2097	2100
7	$Cys382^3$H	SH^- (CO)	ADT-H	−1	1882	1969	2001	2115	2136	1859	1942	1973	2088	2103
8	$Cys382^3$H	SH^- (CN^-)	ADT-H	−1	1880	1968	2006	2115	2130	1856	1942	1976	2091	2100
9	$Cys382^3$H	SH^- (ADT)	ADT-H	−1	1886	1966	2002	2113	2135	1858	1944	1968	2085	2107
10	$Cys382^3$H	SH^- (CO)	ADT-H_2^+	0	1917	2007	2033	2133	2148	1889	1982	2001	2098	2118
11	$Cys382^3$H	SH^- (CN^-)	ADT-H_2^+	0	1916	2007	2039	2133	2144	1888	1981	2007	2104	2117
12	$Cys382^3$H	SH_2 (down)	ADT-H	0	1910	2011	2023	2131	2134	1886	1984	1995	2103	2107
13	$Cys382^3$H	SH_2 (up, CN^-)	ADT-H	0	1910	2011	2027	2133	2137	1884	1982	1996	2110	2107
14	SH_2 (up)	SH^- (CO)	ADT-H	−1	1883	1987	2004	2121	2137	1856	1959	1975	2091	2106
15	SH_2 (up)	SH^- (CN^-)	ADT-H	−1	1882	1987	2008	2121	2131	1855	1958	1979	2098	2107
16	SH_2 (up)	SH^- (ADT)	ADT-H	−1	1883	1985	2000	2120	2135	1856	1959	1974	2096	2105
17	SH_2 (down)	SH^- (CO)	ADT-H	−1	1882	1977	2002	2114	2137	1856	1956	1975	2087	2107
18	SH_2 (down)	SH^- (CN^-)	ADT-H	−1	1880	1977	2006	2113	2131	1858	1945	1973	2083	2104
19	SH_2 (down)	SH^- (ADT)	ADT-H	−1	1882	1976	1998	2116	2135	1856	1948	1968	2082	2104
20	SH^- (CO)	SH^- (CO)	ADT-H	−2	1842	1953	1966	2115	2117	1821	1924	1937	2087	2090
21	SH^- (CO)	SH^- (CN^-)	ADT-H	−2	1841	1954	1967	2110	2117	1819	1928	1941	2085	2092
22	SH^- (CN^-)	SH^- (CO)	ADT-H	−2	1840	1953	1968	2110	2115	1818	1923	1937	2082	2089
23	SH^- (CN^-)	SH^- (CN^-)	ADT-H	−2	1839	1955	1968	2109	2111	1814	1929	1942	2082	2083

‡For all computational models, the chemical nature of the ligand bound to the distal iron (Fe_d) and the proximal iron (Fe_p) is indicated. For –SH^- and –SH_2 ligands, the orientation relative to other ligands is indicated in parentheses. Harmonic frequencies ω^h and anharmonic frequencies ω^a are listed for all CX stretch modes in units of cm^{-1}. See Fig. S7 for graphical representations.

Atomic positions (Cartesian coordinates in units of Å) of the computational models utilized in this study. Models are numbered according to Table S1.

Model 1 (Cys382^{-}, SH^{-} (CO), ADT-H): 32 atoms, Charge = −2, Multiplicity = 1

Fe	-9.873745562	-18.88946623	23.97179735
Fe	-9.510233562	-16.79261735	25.34692237
S	-11.57578524	-17.825193	25.19386712
S	-9.467254784	-16.81388534	23.00049801
O	-7.762138432	-20.17876651	22.40071959
N	-10.56104577	-21.51395229	25.48796345
O	-7.68116119	-19.07385415	25.95137436
N	-6.651047402	-15.5626825	25.23328112
O	-9.495130146	-16.97065791	28.27026455
C	-8.595992436	-19.64593361	23.03962425
C	-10.29676806	-20.50072785	24.92775976
C	-8.573795639	-18.55203926	25.36154605
C	-7.736265006	-16.03997604	25.27322848
C	-9.501465774	-16.9134006	27.09393189
C	-11.08918967	-16.14706408	22.30382179
N	-12.13045317	-15.78883804	23.23997751
C	-12.68066036	-16.91257068	23.96255958
H	-11.4650579	-16.90899308	21.59516145
H	-10.79357583	-15.24420497	21.73902559
H	-11.71249727	-15.13112582	23.93056818
H	-13.01612791	-17.68953013	23.24911467
H	-13.54285762	-16.5675313	24.56231972
C	-11.29919902	-21.42336653	22.08803296
S	-11.26842149	-19.57931593	22.18139922
H	-11.45266848	-21.82320414	23.10453491
H	-10.31587263	-21.78980256	21.73485497
C	-12.4053893	-21.89129947	21.13328264
H	-12.42488147	-22.99850028	21.05700646
H	-13.39687829	-21.55314875	21.48736453
H	-12.25890201	-21.48146027	20.11547919
S	-10.26073769	-14.55787898	25.72596403
H	-11.40547346	-14.84022549	26.42101597

Model 2 (Cys382⁻, SH⁻ (CN⁻), ADT-H): 32 atoms, Charge = −2, Multiplicity = 1

Fe	-9.884150342	-18.88757045	23.96871951
Fe	-9.516559107	-16.7851022	25.33715957
S	-11.5872477	-17.81265996	25.17912692
S	-9.469440203	-16.81580705	22.9903151
O	-7.772050519	-20.18850572	22.40680601
N	-10.58199582	-21.50385918	25.49389463
O	-7.692331028	-19.0687686	25.94891898
N	-6.676303706	-15.52651396	25.23600492
O	-9.511224214	-16.96090793	28.26173064
C	-8.605970597	-19.65139104	23.04232008
C	-10.31474552	-20.49311144	24.9306289
C	-8.585862869	-18.5487257	25.35829862
C	-7.748134485	-16.03537017	25.27076443
C	-9.516890898	-16.9029458	27.08625808
C	-11.09040052	-16.15626849	22.28132865
N	-12.13800696	-15.7913886	23.20653604
C	-12.68987998	-16.90615613	23.94123085
H	-11.45940073	-16.92620529	21.57772244
H	-10.79341653	-15.2585301	21.70890007
H	-11.73257373	-15.11938754	23.88899938
H	-13.0302419	-17.68861848	23.23616762
H	-13.54763391	-16.5508909	24.54088647
C	-11.29374479	-21.42885047	22.08538366
S	-11.27727569	-19.58464357	22.1791604
H	-11.44636457	-21.8295002	23.10174144
H	-10.3070539	-21.78800977	21.7340708
C	-12.39470725	-21.90521576	21.12877729
H	-12.40586066	-23.01255892	21.05269931
H	-13.38927362	-21.574349	21.48106652
H	-12.24946565	-21.4944968	20.11113423
S	-10.313297	-14.5614897	25.70153005
H	-9.196113782	-13.89885294	25.28785269

Model 3 ($Cys382^-$, SH^- (ADT), ADT-H): 32 atoms, Charge = −2, Multiplicity = 1

Fe	-9.945135863	-18.88189981	23.95588508
Fe	-9.50188249	-16.78148206	25.31363669
S	-11.6089592	-17.77841545	25.20581905
S	-9.54382198	-16.79818913	22.98733754
O	-7.825991407	-20.18019927	22.40724917
N	-10.66390503	-21.51206406	25.44275907
O	-7.812037473	-19.13266403	26.00194471
N	-6.560794405	-15.78355262	25.17594975
O	-9.537631167	-16.82065353	28.24268915
C	-8.661831932	-19.64115486	23.03989541
C	-10.38477477	-20.49503273	24.89660943
C	-8.660037643	-18.56302707	25.39085125
C	-7.686273328	-16.15536162	25.22299104
C	-9.522777917	-16.81391578	27.06576716
C	-11.1469144	-16.16635167	22.30106019
N	-12.16686426	-15.81924753	23.29158193
C	-12.74318645	-16.96883311	23.98901448
H	-11.48527883	-16.93912576	21.57185341
H	-10.89310458	-15.24178784	21.75048053
H	-12.92636318	-15.31922238	22.80594597
H	-13.0668384	-17.78651083	23.30349324
H	-13.61476842	-16.60676545	24.56540416
C	-11.33013895	-21.40130482	22.03033647
H	-11.45658781	-21.83537007	23.03669685
H	-10.33911243	-21.72067368	21.65413464
C	-12.43165949	-21.88049333	21.07597189
H	-12.41324716	-22.98496784	20.96643354
H	-13.4296941	-21.58891735	21.45270926
H	-12.31387705	-21.43563424	20.06907699
S	-10.09997534	-14.48688125	25.62656002
H	-11.20558873	-14.65050073	24.83074348
S	-11.36382311	-19.56060972	22.18256398

Model 4 (Cys382$^-$, SH_2 (down), ADT-H): 33 atoms, Charge = −1, Multiplicity = 1

Fe	-9.55634877	-18.86645568	23.73093351
Fe	-9.436372658	-16.81255063	25.20219182
S	-11.37603279	-18.06372819	24.99956416
S	-9.392545454	-16.69611289	22.87437165
O	-7.328504037	-19.80587352	22.05740887
N	-9.892775	-21.58884829	25.18006776
O	-7.321070092	-18.93549867	25.6443995
N	-6.796401253	-15.19968711	25.3106271
O	-9.326503406	-17.25625444	28.10054877
C	-8.215672657	-19.4309432	22.71991419
C	-9.76898965	-20.54792241	24.62788064
C	-8.279175168	-18.5626867	25.05929007
C	-7.745162967	-15.90714642	25.22974297
C	-9.362992432	-17.11678865	26.93560332
C	-11.08469612	-16.24738696	22.15586969
N	-12.21385153	-16.11224314	23.04435812
C	-12.58451817	-17.30986291	23.75804106
H	-11.30288355	-17.04841717	21.42707726
H	-10.92552791	-15.29206792	21.62741374
H	-12.06839771	-15.32971469	23.69273573
H	-12.75823461	-18.11381679	23.01955403
H	-13.50711502	-17.1191925	24.33315361
C	-11.96181434	-21.01591637	22.55726172
H	-12.64001114	-20.59275932	23.3220356
H	-11.35859278	-21.7874425	23.061691
C	-12.75386491	-21.5992173	21.380658
H	-13.44108861	-22.39191464	21.73524968
H	-13.35976865	-20.82606287	20.87287976
H	-12.07629285	-22.04171738	20.62873312
S	-10.82198709	-19.69253009	21.95248467
S	-10.32028127	-14.71917672	25.49797722
H	-10.91766397	-14.68178597	26.72917662
H	-9.224643154	-14.01268893	25.92433965

Model 5 (Cys382^{-}, SH_2 (up, CN^{-}), ADT-H): 33 atoms, Charge = −1, Multiplicity = 1

Fe	-9.5738189	-18.87199567	23.84424494
Fe	-9.5256502	-16.85998615	25.37214426
S	-11.45489108	-18.08419837	25.03244735
S	-9.358650705	-16.67517672	23.05630982
O	-7.266248471	-19.78649458	22.26675222
N	-9.9993455	-21.62500457	25.20521442
O	-7.436866961	-18.99806758	25.8621003
N	-6.822452026	-15.33249421	25.54982997
O	-9.56848619	-17.5146935	28.24044694
C	-8.184721581	-19.42175345	22.89079542
C	-9.84184725	-20.5716144	24.68635843
C	-8.364059309	-18.60791237	25.2399813
C	-7.809377363	-15.98556296	25.47551623
C	-9.55111233	-17.26373219	27.09558329
C	-11.00335579	-16.22499212	22.24680543
N	-12.17810892	-16.09160908	23.07876733
C	-12.59211035	-17.30524932	23.74676177
H	-11.18938149	-17.02140325	21.50415542
H	-10.81797932	-15.26822022	21.72847202
H	-12.02821547	-15.34847959	23.7710055
H	-12.73130333	-18.08919737	22.98015705
H	-13.54418504	-17.12179514	24.27420194
C	-11.92266601	-20.98107672	22.49424743
H	-12.63510479	-20.57355211	23.23589695
H	-11.34955325	-21.77001291	23.00669265
C	-12.65867736	-21.52700956	21.26457097
H	-13.36657221	-22.32446567	21.56329969
H	-13.23482049	-20.73651087	20.74893064
H	-11.94722994	-21.95353313	20.53505062
S	-10.74629262	-19.65020058	21.98243209
S	-10.1601826	-14.70854947	25.86710254
H	-9.74135461	-13.99757148	24.77890182
H	-11.48564756	-14.46673069	25.58521924

Model 6 (Cys382$^-$, SH$^-$ (CN$^-$), ADT-H_2^+): 33 atoms, Charge = −1, Multiplicity = 1

Fe	-9.60672446	-18.85827682	23.81907599
Fe	-9.6225492	-16.86604088	25.36606196
S	-11.49470185	-18.19643686	25.04977061
S	-9.424532459	-16.66960852	23.03727832
O	-7.251864338	-19.75289727	22.31525665
N	-9.90892823	-21.64687477	25.13183226
O	-7.463101197	-18.88207892	25.86950287
N	-7.03947252	-15.16755351	25.51623887
O	-9.61714192	-17.26565721	28.26839517
C	-8.184629269	-19.38245219	22.91585176
C	-9.78957679	-20.57617458	24.63794766
C	-8.404456019	-18.47369591	25.27943154
C	-8.007456066	-15.8490744	25.46785441
C	-9.62483821	-17.12770021	27.10587813
C	-11.03738483	-16.20249572	22.30408183
N	-12.12624647	-16.07575627	23.33238645
C	-12.6348495	-17.38828364	23.86422409
H	-11.3484792	-16.96749715	21.56805645
H	-10.93245353	-15.21257165	21.82898428
H	-12.91631492	-15.54127231	22.93235733
H	-12.78322196	-18.06412983	22.99947983
H	-13.5872139	-17.17641594	24.37812464
C	-11.92056862	-20.98260001	22.44400451
H	-12.64676095	-20.62684481	23.19961737
H	-11.30547169	-21.7510057	22.93896542
C	-12.64009302	-21.54461168	21.21222607
H	-13.30780092	-22.37933481	21.50185252
H	-13.25673556	-20.77450442	20.71172831
H	-11.91457668	-21.92475197	20.47118051
S	-10.80673565	-19.58958655	21.95381399
S	-10.80718858	-14.83730868	25.75458128
H	-11.70343165	-15.48249174	24.20661117
H	-9.91117171	-13.9929475	25.16921933

Model 7 (Cys382^{3}H, SH$^-$ (CO), ADT-H): 33 atoms, Charge = −1, Multiplicity = 1

Fe	-9.994553	-18.95123053	23.90890923
Fe	-9.420729444	-16.89699602	25.24849211
S	-11.5564252	-17.78362162	25.20072377
S	-9.50803089	-16.93082629	22.89651214
O	-7.845888648	-20.3300522	22.46032547
N	-10.78907897	-21.5360202	25.44089133
O	-7.772864698	-19.25052178	25.904752
N	-6.497153935	-15.90065562	24.89556034
O	-9.334784758	-16.93029132	28.1830435
C	-8.681151056	-19.75952379	23.05733119
C	-10.44733505	-20.52672247	24.9202151
C	-8.597222523	-18.6010135	25.35743719
C	-7.60989675	-16.27782908	25.03964961
C	-9.368960176	-16.9178919	27.01372561
C	-11.1079513	-16.08519157	22.3182056
N	-12.0432881	-15.65326909	23.32052302
C	-12.65353598	-16.71214387	24.0777146
H	-11.61185513	-16.76531948	21.60471472
H	-10.74494266	-15.20418056	21.76214366
H	-11.54048626	-15.01865356	23.97341886
H	-13.17361475	-17.41156159	23.39675379
H	-13.3961229	-16.28054537	24.77047525
C	-11.3772356	-21.46754531	22.05932106
H	-11.54740832	-21.91362169	23.05352994
H	-10.32807562	-21.66600154	21.78517992
C	-12.36523261	-21.95699577	21.00080922
H	-12.26106862	-23.05081593	20.87588593
H	-13.40797474	-21.7448161	21.29430893
H	-12.18382728	-21.4891992	20.01572573
S	-9.91874914	-14.62140047	25.64370741
H	-11.03417226	-14.7735236	26.42143998
S	-11.54011033	-19.64378148	22.38291155
H	-11.03313009	-19.25435557	21.17118253

Model 8 (Cys382^{3}H, SH^- (CN^-), ADT-H): 33 atoms, Charge = −1, Multiplicity = 1

Fe	-10.02972088	-18.91443324	23.92432406
Fe	-9.523856505	-16.84320078	25.26839319
S	-11.63753542	-17.7831142	25.19258422
S	-9.583391492	-16.88280692	22.91457439
O	-7.827080701	-20.23961763	22.50631774
N	-10.77717077	-21.51540695	25.45244274
O	-7.823963469	-19.1531099	25.94669663
N	-6.646302945	-15.72138383	24.9751736
O	-9.479449207	-16.87875125	28.20572733
C	-8.684894801	-19.69032836	23.09150197
C	-10.4554298	-20.49842689	24.93394621
C	-8.660738153	-18.52647291	25.39099681
C	-7.736933621	-16.16840321	25.09242721
C	-9.500531407	-16.86228922	27.037121
C	-11.20111634	-16.08988312	22.30796426
N	-12.16709189	-15.68052663	23.28943324
C	-12.75296312	-16.74824433	24.05323562
H	-11.67026016	-16.79162252	21.59172492
H	-10.85786596	-15.20225739	21.74981584
H	-11.70694591	-15.01240679	23.93735059
H	-13.24995628	-17.46783649	23.37593977
H	-13.50904434	-16.32879002	24.73842494
C	-11.32179476	-21.47012314	22.06435825
H	-11.49213256	-21.91857335	23.05747281
H	-10.2644382	-21.64101967	21.80343402
C	-12.28346308	-21.98790155	20.99512099
H	-12.14868198	-23.0787958	20.87389577
H	-13.33495328	-21.8030488	21.27559711
H	-12.10276486	-21.51731552	20.01121549
S	-10.16992114	-14.60290911	25.67239609
H	-9.009529733	-14.00381121	25.28563281
S	-11.53747632	-19.65074134	22.3816583
H	-11.02705359	-19.25072126	21.17479546

Model 9 (Cys382^{3}H, SH^- (ADT), ADT-H): 33 atoms, Charge = −1, Multiplicity = 1

Fe	-10.0661131	-18.91285017	23.92282893
Fe	-9.489966258	-16.83384213	25.22477867
S	-11.63964185	-17.76012562	25.21656749
S	-9.644504888	-16.87637163	22.90183104
O	-7.827841132	-20.22623463	22.55358859
N	-10.83107775	-21.52466851	25.41777857
O	-7.967333263	-19.20075158	26.07176395
N	-6.512933723	-16.03808559	24.8649242
O	-9.5325263	-16.62593641	28.15680883
C	-8.701615371	-19.67917134	23.11759355
C	-10.49670091	-20.5025613	24.91727075
C	-8.733831985	-18.52979775	25.46946202
C	-7.65319102	-16.32134153	25.0107295
C	-9.512370639	-16.68857242	26.98887321
C	-11.24554778	-16.10179861	22.33921555
N	-12.17449224	-15.72960701	23.39113187
C	-12.79366837	-16.84221693	24.08768536
H	-11.69007886	-16.7996486	21.5866856
H	-10.94113643	-15.18132526	21.81198788
H	-12.89371195	-15.10006003	23.01307936
H	-13.2444064	-17.60349991	23.40734043
H	-13.59329508	-16.4419806	24.73518554
C	-11.28333554	-21.47175838	22.02531503
H	-11.43646988	-21.94489587	23.00982465
H	-10.22215266	-21.60235647	21.75705561
C	-12.23219494	-21.99799022	20.94886359
H	-12.06280705	-23.08114699	20.80382606
H	-13.28798973	-21.8535756	21.23708928
H	-12.07112326	-21.50126043	19.97436166
S	-9.845049421	-14.50968279	25.50913905
H	-11.11031632	-14.60741154	24.9959148
S	-11.55679552	-19.66668692	22.38231041
H	-11.0757117	-19.22431786	21.17742952

Model 10 (Cys382^{3}H, SH^- (CO), ADT-H_2^+): 34 atoms, Charge = 0, Multiplicity = 1

Fe	-9.994983895	-18.87782623	23.8570663
Fe	-9.528249764	-16.84203	25.25268176
S	-11.62567521	-17.84455728	25.15820729
S	-9.560119679	-16.8263449	22.89891213
O	-7.787122851	-20.21554968	22.45387929
N	-10.66458456	-21.44481277	25.45668999
O	-7.738694575	-19.12091486	25.8327466
N	-6.659669928	-15.71098619	24.96804974
O	-9.402531115	-16.93537672	28.18599344
C	-8.638522587	-19.65787031	23.02619544
C	-10.36539141	-20.45346379	24.882787
C	-8.598227715	-18.50697632	25.31303906
C	-7.7525302	-16.14284063	25.09885473
C	-9.456261161	-16.9148555	27.02326041
C	-11.11540416	-16.01349268	22.32979652
N	-12.04382534	-15.65153001	23.44840467
C	-12.72963675	-16.81103343	24.10701356
H	-11.66467074	-16.64318848	21.60718493
H	-10.82374345	-15.06935979	21.84133035
H	-11.40731801	-15.12480985	24.24519752
H	-13.20526033	-17.43102343	23.3260987
H	-13.5097949	-16.39024823	24.76217844
C	-11.44913344	-21.50385648	22.1501257
H	-11.79550289	-21.8361393	23.14163054
H	-10.39803675	-21.81794659	22.0479512
C	-12.33193198	-21.98840235	21.00201
H	-12.32595666	-23.09312753	20.98961762
H	-13.37733052	-21.65622626	21.12144649
H	-11.96516976	-21.64273629	20.01902103
S	-10.18235914	-14.59475977	25.57004216
H	-11.14104757	-14.75805121	26.53075532
S	-11.4569566	-19.64462641	22.28644109
H	-10.82884701	-19.41651927	21.09265476
H	-12.75054948	-14.97632298	23.10765257

Model 11 (Cys382[3]H, SH^- (CN^-), ADT-H_2^+): 34 atoms, Charge = 0, Multiplicity = 1

Fe	-10.01912375	-18.85392188	23.87613904
Fe	-9.624899552	-16.8367986	25.32331128
S	-11.68847244	-17.89332468	25.18288344
S	-9.635898522	-16.77116705	22.96437283
O	-7.76935744	-20.10682154	22.46152464
N	-10.61769013	-21.46276407	25.43519766
O	-7.744918056	-19.05602988	25.84053555
N	-6.838476888	-15.51309874	25.10627023
O	-9.524312978	-17.03242281	28.25528868
C	-8.637965341	-19.58240129	23.03973347
C	-10.34732143	-20.4547597	24.87613954
C	-8.631502404	-18.4627689	25.34226701
C	-7.890743115	-16.04302892	25.2117615
C	-9.569978833	-16.9674198	27.09511622
C	-11.21337866	-16.00762769	22.38936584
N	-12.15527692	-15.67725709	23.50559438
C	-12.81401744	-16.85853179	24.15513668
H	-11.73983855	-16.65451361	21.66501008
H	-10.95240306	-15.05472834	21.90049256
H	-12.88105981	-15.02122752	23.16731612
H	-13.28678628	-17.4747383	23.3695028
H	-13.59271044	-16.45882646	24.82504309
C	-11.39819862	-21.48990223	22.12486966
H	-11.741102	-21.84261065	23.11051277
H	-10.3396792	-21.77843313	22.02390753
C	-12.26445352	-21.98037655	20.96672408
H	-12.23252142	-23.08441519	20.94035565
H	-13.31784896	-21.67430307	21.08560634
H	-11.90175709	-21.61366704	19.98985664
S	-11.44967024	-19.63309967	22.28421497
H	-10.81822789	-19.37593823	21.09803158
S	-10.42142098	-14.64985311	25.73489439
H	-11.54433528	-15.13970568	24.31314136
H	-9.445193632	-13.97386323	25.06614995

Model 12 (Cys382[3]H, SH_2 (down), ADT-H): 34 atoms, Charge = 0, Multiplicity = 1

Fe	-9.567551371	-18.82973949	23.6565642
Fe	-9.469260649	-16.85876124	25.19667069
S	-11.39497876	-18.11797837	24.92541633
S	-9.449023786	-16.63848465	22.87974847
O	-7.177652268	-19.59812138	22.11408612
N	-9.833930556	-21.63856182	24.93915169
O	-7.406876115	-18.96386064	25.67356387
N	-6.795245536	-15.30672153	25.23970483
O	-9.438877417	-17.3524393	28.09889953
C	-8.109085561	-19.27863888	22.73795918
C	-9.704758367	-20.55192777	24.48741036
C	-8.331785323	-18.50711868	25.10391605
C	-7.766209625	-15.98397817	25.2088146
C	-9.44171457	-17.19000898	26.94391429
C	-11.15827577	-16.12953211	22.20670231
N	-12.2625357	-16.02759601	23.11801056
C	-12.65055887	-17.22703953	23.79875245
H	-11.39620832	-16.86405605	21.41768931
H	-10.98141538	-15.14937001	21.73569707
H	-12.1460834	-15.24366206	23.76717075
H	-12.97721495	-17.97855123	23.05748986
H	-13.49964906	-17.01014619	24.46737013
C	-12.06956905	-20.98769953	22.51840388
H	-12.82841335	-20.4470838	23.10958049
H	-11.50405322	-21.63412475	23.20931457
C	-12.68199297	-21.73234354	21.3328861
H	-13.39967894	-22.48017392	21.71540277
H	-13.22309994	-21.05851096	20.64650816
H	-11.91473907	-22.27754465	20.75513366
S	-10.90185394	-19.65267391	21.95008791
S	-10.29752878	-14.73357441	25.56362411
H	-10.46026748	-14.60911966	26.91341017
H	-9.120922228	-14.02907511	25.56703569
H	-10.07252516	-20.48853267	21.26169251

Model 13 (Cys382^{3}H, SH_2 (up, CN^-), ADT-H): 34 atoms, Charge = 0, Multiplicity = 1

Fe	-10.0011948	-18.8476208	23.97717365
Fe	-9.527577989	-16.80887954	25.34542923
S	-11.63408279	-17.74689577	25.24933378
S	-9.585795472	-16.77932256	23.02207689
O	-7.815780545	-20.11886596	22.4647936
N	-10.79631799	-21.44745415	25.47068576
O	-7.839266318	-19.19538362	25.96072061
N	-6.589663067	-15.82954059	25.10347447
O	-9.499496082	-17.18413631	28.27370103
C	-8.663992741	-19.60299163	23.07724019
C	-10.45880757	-20.44329386	24.94304926
C	-8.69046534	-18.63524745	25.36884125
C	-7.684525136	-16.26798341	25.20588601
C	-9.505278272	-17.04315309	27.1177207
C	-11.2274075	-16.06360215	22.36503903
N	-12.26849039	-15.72850633	23.29461743
C	-12.79503465	-16.81138878	24.08238848
H	-11.61656982	-16.79559197	21.63510804
H	-10.92418819	-15.15873199	21.81349317
H	-11.98654762	-14.94477184	23.89145223
H	-13.22774698	-17.57455119	23.41174016
H	-13.59558196	-16.43008005	24.73709732
C	-11.34148016	-21.4442521	22.11912158
H	-11.61935811	-21.87012878	23.09651003
H	-10.27580525	-21.66654512	21.94841048
C	-12.23717016	-21.91630797	20.97515848
H	-12.14764865	-23.01369834	20.88403753
H	-13.29769435	-21.67655978	21.16183724
H	-11.94404827	-21.4762898	20.00539405
S	-11.49627467	-19.60776925	22.39048658
H	-10.92941181	-19.24295443	21.20070418
S	-9.66330239	-14.53925106	25.73365388
H	-9.196082563	-14.0145448	24.56561877
H	-10.93759339	-14.07550456	25.51910591

Model 14 (SH_2 (up), SH^- (CO), ADT-H): 27 atoms, Charge = −1, Multiplicity = 1

Fe	-9.996421265	-18.88596141	23.88729909
Fe	-9.526961857	-16.8467851	25.27709807
S	-11.63764882	-17.78732241	25.13365812
S	-9.53134689	-16.83389399	22.9266401
O	-7.706168309	-20.12658171	22.51796364
N	-10.76856614	-21.51071527	25.36546948
O	-7.84249163	-19.1700643	25.95111835
N	-6.622576138	-15.77075144	25.04213787
O	-9.526221127	-16.94248573	28.21341707
C	-8.610665188	-19.62400476	23.06668969
C	-10.42018542	-20.49566055	24.86241982
C	-8.664528885	-18.53700088	25.38184226
C	-7.728897631	-16.17972424	25.14098772
C	-9.526410926	-16.90661576	27.04445879
C	-11.12256499	-16.01747142	22.30855636
N	-12.09870636	-15.61418812	23.28634013
C	-12.71280406	-16.69303528	24.00930115
H	-11.59229855	-16.6960794	21.5679443
H	-10.76430342	-15.12459604	21.76858876
H	-11.6255235	-14.98411636	23.96668271
H	-13.22636845	-17.37228699	23.30085471
H	-13.47184584	-16.282338	24.6969425
S	-10.09263559	-14.59498451	25.7051652
H	-11.22466175	-14.79036434	26.44868489
S	-11.45295421	-19.80170326	22.37641022
H	-12.53265842	-20.08911887	23.16167231
H	-12.11083966	-18.77311029	21.74740989

Model 15 (SH_2 (up), SH^- (CN^-), ADT-H): 27 atoms, Charge = −1, Multiplicity = 1

Fe	-10.00031666	-18.88611733	23.88245058
Fe	-9.528964927	-16.83813049	25.26192986
S	-11.64231907	-17.7740421	25.11634742
S	-9.530193907	-16.84005914	22.90992605
O	-7.70855129	-20.13901807	22.52622341
N	-10.77847762	-21.49866981	25.37862143
O	-7.84636274	-19.15883385	25.9489981
N	-6.648098988	-15.71262729	25.05239437
O	-9.539643554	-16.92645719	28.20028876
C	-8.613953418	-19.63209832	23.06983559
C	-10.42843232	-20.48741701	24.86908539
C	-8.670056488	-18.52891193	25.37804285
C	-7.740438342	-16.16252416	25.13717287
C	-9.538800932	-16.89027481	27.03231524
C	-11.1219726	-16.03567568	22.27521856
N	-12.10663809	-15.62336985	23.23962531
C	-12.71809811	-16.69057963	23.98166346
H	-11.58317692	-16.72654799	21.54067029
H	-10.76480184	-15.14885898	21.72456966
H	-11.6507761	-14.96970696	23.90653778
H	-13.23637901	-17.37974646	23.28617642
H	-13.4708855	-16.26722661	24.66811352
S	-10.18057448	-14.60619908	25.69449603
H	-9.014199588	-14.00107319	25.3361677
S	-11.45525303	-19.81153035	22.37699552
H	-12.53495254	-20.09618976	23.16330634
H	-12.1150034	-18.78818571	21.74133873

Model 16 (SH_2 (up), SH^- (ADT), ADT-H): 27 atoms, Charge = −1, Multiplicity = 1

Fe	-10.00356605	-18.88432724	23.86476303
Fe	-9.49434333	-16.82210722	25.20470653
S	-11.62783098	-17.74320379	25.11017258
S	-9.554380867	-16.84413179	22.85861941
O	-7.68144362	-20.13571548	22.56672577
N	-10.81002119	-21.4974565	25.34124041
O	-7.947641204	-19.17980412	26.0320249
N	-6.541132541	-15.91430114	24.89732287
O	-9.540787968	-16.74549472	28.14511388
C	-8.600279927	-19.62703056	23.08639206
C	-10.44524021	-20.48553206	24.84321058
C	-8.718865774	-18.52570848	25.4161725
C	-7.667032506	-16.25725953	25.02483175
C	-9.519456229	-16.74877207	26.97500544
C	-11.13802267	-16.07973727	22.24547067
N	-12.0711769	-15.69150601	23.27419469
C	-12.73044504	-16.76996703	23.96344834
H	-11.57186861	-16.78979517	21.49136521
H	-10.82139232	-15.17324601	21.70189866
H	-12.73812314	-14.99123712	22.93395155
H	-13.21237754	-17.51908467	23.28356427
H	-13.52338148	-16.34703501	24.60480847
S	-9.861820328	-14.54427462	25.62325158
H	-11.20559397	-14.62160235	25.85625196
S	-11.43115131	-19.82458205	22.34707423
H	-12.54414472	-20.06300414	23.10241438
H	-12.04520748	-18.7986143	21.66653216

Model 17 (SH_2 (down), SH^- (CO), ADT-H): 27 atoms, Charge = −1, Multiplicity = 1

Fe	-9.980608537	-18.78113633	23.78794472
Fe	-9.5321312	-16.81623416	25.28827197
S	-11.63508777	-17.7690244	25.09720779
S	-9.536171218	-16.66994625	22.94164675
O	-7.757593589	-19.98293793	22.2950322
N	-10.74355257	-21.52368366	25.00975355
O	-7.816202084	-19.15194362	25.82634493
N	-6.63685971	-15.70072716	25.11614686
O	-9.53805759	-17.08139968	28.2141685
C	-8.618863639	-19.48273764	22.91491673
C	-10.38980963	-20.45323371	24.64084757
C	-8.648407212	-18.49742718	25.29688
C	-7.739695851	-16.12408098	25.19101346
C	-9.535226966	-16.97750411	27.04908515
C	-11.14629285	-15.84990109	22.36334373
N	-12.12100914	-15.50960865	23.36535332
C	-12.72752079	-16.63006351	24.0271165
H	-11.60460908	-16.50807038	21.60136204
H	-10.80196985	-14.92563529	21.86912204
H	-11.65611949	-14.90933798	24.07600566
H	-13.22586023	-17.28052258	23.28506467
H	-13.48768869	-16.2639977	24.73819606
S	-10.11868958	-14.59858763	25.8436178
H	-11.25047023	-14.84750958	26.57135796
S	-11.51353214	-19.42798377	22.20372775
H	-10.80835845	-19.94085693	21.15044893
H	-11.83287053	-20.67573587	22.67676844

Model 18 (SH_2 (down), SH^- (CN^-), ADT-H): 27 atoms, Charge = −1, Multiplicity = 1

Fe	-9.97272067	-18.783329	23.7849861
Fe	-9.508134227	-16.82854952	25.29629281
S	-11.61687816	-17.77176222	25.10842165
S	-9.527402693	-16.66703896	22.94977018
O	-7.761193334	-19.98281198	22.27270851
N	-10.74001486	-21.52958181	24.99511588
O	-7.795952556	-19.17307931	25.80729826
N	-6.634697918	-15.67203521	25.14277189
O	-9.502892151	-17.12114731	28.22148747
C	-8.617807346	-19.48403623	22.90050419
C	-10.38387425	-20.45837241	24.63067681
C	-8.632087221	-18.5136867	25.28952385
C	-7.724186796	-16.13254266	25.20483662
C	-9.507975121	-17.00336282	27.05870897
C	-11.14209466	-15.84912687	22.37762902
N	-12.11670424	-15.51032813	23.37925588
C	-12.71704211	-16.62810291	24.05082619
H	-11.59896513	-16.50972202	21.61697044
H	-10.80164818	-14.92467279	21.88099621
H	-11.66252878	-14.89603746	24.08224234
H	-13.22663263	-17.27880141	23.31652241
H	-13.46497418	-16.2575768	24.77212358
S	-10.1699708	-14.63651987	25.88583178
H	-9.011540004	-13.9989887	25.55931535
S	-11.51669848	-19.41597424	22.20596717
H	-10.81825955	-19.94213924	21.15482742
H	-11.84960379	-20.65826557	22.68445888

Model 19 (SH_2 (down), SH^- (ADT), ADT-H): 27 atoms, Charge = −1, Multiplicity = 1

Fe	-10.01518509	-18.79742553	23.79155691
Fe	-9.499180719	-16.7871392	25.20942627
S	-11.62748812	-17.71863884	25.10983358
S	-9.588685615	-16.71353311	22.86676965
O	-7.755297985	-20.03364266	22.38644407
N	-10.77118317	-21.52716765	25.04986136
O	-7.940042565	-19.17065368	25.93175093
N	-6.546995158	-15.86820752	24.90726043
O	-9.530732112	-16.81535233	28.15076469
C	-8.636713839	-19.51963202	22.96601339
C	-10.43377986	-20.4560232	24.66873142
C	-8.717069875	-18.49133001	25.35140092
C	-7.672329973	-16.21379721	25.03307839
C	-9.514854321	-16.77562595	26.98108265
C	-11.19207407	-15.94377731	22.29987974
N	-12.11780813	-15.61069191	23.35678873
C	-12.75822976	-16.73054855	24.00277634
H	-11.61876669	-16.63096169	21.52621614
H	-10.89114931	-15.00823405	21.79781227
H	-12.80255773	-14.9142585	23.04415174
H	-13.21765287	-17.46494029	23.29819086
H	-13.55555097	-16.34631346	24.66286264
S	-9.864131789	-14.52477892	25.71281037
H	-11.21275969	-14.61054991	25.90979417
S	-11.50743088	-19.50530692	22.21176622
H	-10.84305456	-19.57914435	21.01699987
H	-11.49284038	-20.86259662	22.39248519

Model 20 (SH⁻ (CO), SH⁻ (CO), ADT-H): 26 atoms, Charge = −2, Multiplicity = 1

Fe	-10.00231646	-18.94581848	24.04216307
Fe	-9.428734931	-16.82849952	25.29864255
S	-11.56523528	-17.72421787	25.30770123
S	-9.524571404	-16.94403775	22.95320273
O	-8.001330367	-20.4049471	22.47354529
N	-10.76532453	-21.43833525	25.7496868
O	-7.735086297	-19.19932936	25.93050877
N	-6.503311252	-15.81142156	24.97049895
O	-9.266552541	-16.8735757	28.22210185
C	-8.796122538	-19.81327107	23.10892374
C	-10.47330449	-20.48621878	25.10552781
C	-8.618164413	-18.64350151	25.35789511
C	-7.614525726	-16.20792998	25.09310958
C	-9.332752822	-16.8696112	27.04643381
C	-11.13932672	-16.19108834	22.33049459
C	-12.67427896	-16.79033956	24.09920835
H	-11.61570423	-16.95631339	21.68983169
H	-10.82003521	-15.33503165	21.70832248
H	-13.0833184	-17.56829508	23.42691201
H	-13.48532421	-16.37444997	24.72510268
H	-11.11812103	-14.70212105	26.38980359
H	-10.99826894	-19.21081089	21.32007009
S	-9.999090494	-14.53030136	25.6205631
S	-11.53330096	-19.78535918	22.44135253
N	-12.09426727	-15.72732344	23.30996402
H	-11.59272367	-15.07777756	23.95091906

Model 21 (SH^- (CO), SH^- (CN^-), ADT-H): 26 atoms, Charge = −2, Multiplicity = 1

Fe	-10.01534862	-18.94955133	24.04031677
Fe	-9.51681681	-16.83828133	25.34186938
S	-11.63587633	-17.77959575	25.28159448
S	-9.555124937	-16.92080659	22.9928269
O	-7.944001548	-20.34199145	22.50126908
N	-10.76677624	-21.48246641	25.69215169
O	-7.786710656	-19.18265373	25.97586536
N	-6.626130299	-15.71941134	25.10975856
O	-9.427839469	-16.933406	28.26832779
C	-8.767404778	-19.77746142	23.1250156
C	-10.48008347	-20.51452618	25.06953588
C	-8.670620439	-18.63864295	25.3926831
C	-7.718724097	-16.17519042	25.19579753
C	-9.467324889	-16.907474	27.09257707
C	-11.17350386	-16.20095785	22.33710674
C	-12.73900756	-16.85564295	24.06019664
H	-11.61105028	-16.97213965	21.67630555
H	-10.86018393	-15.33072406	21.73155532
H	-13.11858115	-17.63498416	23.37245337
H	-13.56898003	-16.46196575	24.6750714
H	-9.064667703	-13.97234754	25.20214786
H	-10.93708826	-19.19757469	21.28919193
S	-10.19335324	-14.5686331	25.68042626
S	-11.48584115	-19.80087336	22.3885726
N	-12.166863	-15.77269145	23.2930176
H	-11.70756525	-15.10962772	23.94968606

Model 22 (SH^- (CN^-), SH^- (CO), ADT-H): 26 atoms, Charge = −2, Multiplicity = 1

Fe	-10.00584881	-18.84155289	23.91759154
Fe	-9.446521573	-16.75907901	25.23873184
S	-11.58040917	-17.65709425	25.21024372
S	-9.525046691	-16.81075861	22.89092041
O	-7.992396156	-20.25591335	22.32310082
N	-10.81657516	-21.42156869	25.45208996
O	-7.766688046	-19.15502069	25.8284765
N	-6.516146254	-15.74099288	24.96059643
O	-9.30200754	-16.88613654	28.16102955
C	-8.792774621	-19.67985152	22.96432401
C	-10.49440358	-20.42503236	24.89354933
C	-8.63970135	-18.58321487	25.25554657
C	-7.629403283	-16.13764073	25.06298474
C	-9.360702206	-16.84916772	26.98529493
C	-11.13728976	-16.04536414	22.27401805
C	-12.68418531	-16.69574494	24.01805875
H	-11.60441833	-16.78735759	21.60092988
H	-10.81338759	-15.16854481	21.68403302
H	-13.08715574	-17.45832287	23.32474529
H	-13.49938976	-16.29642182	24.6493717
H	-11.1443135	-14.66376205	26.37238703
H	-12.01132034	-20.64419773	22.8377348
S	-10.01937619	-14.4717015	25.61668386
S	-11.50841147	-19.53853271	22.21862659
N	-12.10108975	-15.61240259	23.2595355
H	-11.6060261	-14.97800771	23.92009588

Model 23 (SH^- (CN^-), SH^- (CN^-), ADT-H): 26 atoms, Charge = −2, Multiplicity = 1

Fe	-10.00780583	-18.8420066	23.91555122
Fe	-9.439837672	-16.75317938	25.226131
S	-11.57797973	-17.64831716	25.20423997
S	-9.527311108	-16.81558391	22.87834449
O	-8.000474538	-20.26747961	22.32186409
N	-10.81862832	-21.41313978	25.46429807
O	-7.759338673	-19.14917844	25.81580525
N	-6.527709395	-15.70322285	24.94441073
O	-9.290063474	-16.87637472	28.14935802
C	-8.798459684	-19.68736019	22.96263905
C	-10.49711759	-20.41928324	24.90060523
C	-8.636868189	-18.58027687	25.2462843
C	-7.629323936	-16.13300258	25.04799677
C	-9.353851269	-16.83911884	26.97472407
C	-11.14372744	-16.05841359	22.25802883
C	-12.68773797	-16.6949346	24.01124809
H	-11.60736035	-16.80849095	21.59158304
H	-10.82276411	-15.1862232	21.65940262
H	-13.09783562	-17.46312359	23.32845195
H	-13.49539093	-16.28693239	24.64623552
H	-8.925796143	-13.89740256	25.09780259
H	-12.0116793	-20.65284035	22.84580058
S	-10.05781056	-14.47322737	25.59320294
S	-11.51456521	-19.54700413	22.22240919
N	-12.1093422	-15.61978923	23.23801657
H	-11.62366275	-14.97001935	23.88867415
